# Nine-Week Co-Supplementation with Sugarcane Wax Alcohol (Policosanol), Phosphatidylserine, and *Ginkgo biloba* Leaf Extract Attenuates the Metabolic Dysfunction and Oxidative Stress in Blood and Vital Organs of Hyperlipidemic/Hyperglycemic Zebrafish

**DOI:** 10.3390/ijms27177913

**Published:** 2026-09-04

**Authors:** Kyung-Hyun Cho, Ashutosh Bahuguna, Cheolmin Jeon, Ji-Eun Kim, Sang Hyuk Lee, Yunki Lee, Seung Hee Baek

**Affiliations:** Raydel HDL Research Institute, Medical Innovation Complex, Daegu 41061, Republic of Korea

**Keywords:** antioxidants, body weight, brain, functional food, intestine, oxidative stress, senescence

## Abstract

Policosanol (sugarcane wax alcohol), phosphatidylserine, and *Ginkgo biloba* leaf extract are Ministry of Food and Drug Safety (MFDS), Republic of Korea-listed functional foods for health-promoting effects. Herein, a combination of policosanol, phosphatidylserine, and *G. biloba* leaf extract (hereafter PPG) was investigated against metabolic stress induced by a high-cholesterol, high-galactose (HCHG) diet in hyperlipidemic and hyperglycemic zebrafish. After 9 weeks of feeding, the zebrafish following the HCHG diet co-supplemented with PPG exhibited a significant 14.1% (*p* < 0.02) reduction in body weight and higher zebrafish survivability compared to the HCHG-supplemented group. Significantly reduced total cholesterol (TC, 1.2-fold), triglycerides (TG, 1.4-fold), low-density lipoprotein cholesterol (LDL-C, 1.3-fold), and glucose levels (1.2-fold), along with a 1.5-fold (*p* = 0.003) elevated high-density lipoprotein cholesterol (HDL-C), were observed in the PPG co-supplemented group relative to the HCHG group. In addition, the HCHG-elevated plasma malondialdehyde (MDA), diminished ferric ion reduction activity (FRA), and paraoxonase (PON)-like activity significantly (*p* < 0.001) reverted by 1.6-fold, 1.4-fold, and 1.6-fold, respectively, by co-supplementation with PPG. Consistently, the plasma from the PPG-supplemented group showed greater ability to prevent carboxymethyllysine (CML)-induced apoptotic death, altered heart rate, and developmental deformities in zebrafish embryos. Moreover, PPG exerted significant protective effects against HCHG-induced hepatomegaly and fatty liver, accompanied by marked inhibition of hepatic interleukin (IL)-6 and reactive oxygen species (ROS) generation. Consistently, PPG supplementation inhibits ROS generation and senescence in the liver, intestine, brain, kidney, testis and ovary and protects the organ damage caused by exposure to HCHG. Similarly, in intestinal tissue, HCHG-induced fibrosis and oxidative stress were mitigated by the co-supplementation of PPG. The findings indicate that PPG is an effective combination for preventing dyslipidemia, oxidative stress, inflammation, and multi-organ damage associated with HCHG-mediated metabolic stress in zebrafish.

## 1. Introduction

Sugarcane wax alcohol (policosanol), phosphatidylserine, and *Ginkgo biloba* leaf extract are important functional food components that have been approved by the Ministry of Food and Drug Safety [MFDS, formerly known as Korean Food and Drug administration (KFDA)], Republic of Korea, for various health-promoting activities [1]. In 1990, the authentic policosanol was first extracted from Cuban sugarcane (*Saccharum officinarum* L.) wax [2]. It comprises a characteristic mixture of eight long-chain aliphatic alcohols (LCAA, from C24 to C34) and gained substantial attention as a natural agent for the prevention of dyslipidemia [2]. The composition and abundance of LCAA in policosanol vary substantially depending on the source material’s origin, its geographical location, and the extraction process [2], which ultimately impacts the functionality of policosanol. In addition to policosanol from Cuban sugarcane, several copycat products extracted from various herbal sources, such as rice bran, wheat germ, peanuts, ginseng, and grapes [2], are available with uncertain and variable outcomes. In human studies, Cuban policosanol (Raydel^®^) has been shown to augment cholesterol efflux capacity [3] and affect the quantity and quality of high-density lipoprotein (HDL) [4]. In addition, policosanol demonstrated notable hypotensive and hypoglycemic effects, exhibited antiplatelet aggregation activity, and inhibited the oxidation of low-density lipoprotein (LDL) [5,6,7]. Due to its multiple functions, sugarcane wax-derived policosanol is listed by the MFDS as a helpful agent for dyslipidemia, with a recommended daily intake of 5~20 mg for human consumption [1].

Phosphatidylserine is a major acidic phospholipid and an important component of the mammalian cell membrane; it is especially highly concentrated in the brain and nervous tissue, which plays a key role in cell signaling [8,9]. The PS helps reduce muscle fatigue and soreness, alleviate depression, improve memory, and manage mood, stress, and mental performance [9]. Due to the important nutritional and physiological relevance, phosphatidylserine has been included as a noble food raw material in China since 2010 [9]. Phosphatidylserine occurs naturally in animals, plants, and microorganisms [9]. However, the commonly available source for external supplementation is soy lecithin. Phosphatidylserine has been approved by the MFDS as a functional food, with a recommended daily intake of 300 mg to aid the aging-associated decline in cognitive function and to protect skin from UV radiation [1].

*G. biloba* is the oldest living tree species, having survived for millions of years, and is thus considered a living fossil [10]. *G. biloba* is a rich source of flavonoids, terpenoids, alkylphenols, carboxylic acids, and proanthocyanins [11], and has been part of traditional Chinese medicine for 1000 years as a remedy for lung infections and cardiovascular disease [10]. *G. biloba* leaf extract is widely recognized for its diverse pharmacological properties, including anti-asthmatic [12], wound healing [13], and neuroprotective effects, particularly in neurodegenerative disorders such as Alzheimer’s [14] and Parkinson’s disease [15]. In addition, *G. biloba* has been shown to facilitate cerebral blood flow and have beneficial effects on memory, anxiety, depression, schizophrenia and brain injury through its neuroprotective properties [13,16]. Owing to its broad pharmacological properties, the MFDS listed *G. biloba* for human consumption (36 mg/day) to promote blood circulation and memory enhancement [1].

Although policosanol, phosphatidylserine, and *G. biloba* have been individually explored extensively for their functionality, no studies have yet evaluated the combined effect of these three ingredients. In view of this, a combination of policosanol, phosphatidylserine, and *G. biloba* leaf extract, shortly named as PPG, at the recommended dose suggested by MFDS was prepared and tested to assess the effect of this combination (PPG) on dyslipidemia, hyperglycemia, oxidative stress, and antioxidant variables in hyperlipidemic and hyperglycemic zebrafish. Furthermore, the effect of the combination was assessed on the protection of the liver, kidney, brain, intestine, testis, and ovary in hyperlipidemic and hyperglycemic zebrafish.

Metabolic stress in zebrafish was induced by exposure to a high-cholesterol, high-galactose diet. Excessive intake of galactose has been associated with the formation of galactitol and non-enzymatic advanced glycation end (AGE) products that have detrimental effects on a variety of organs [17]. Accumulation of galactitol impairs cellular antioxidant defenses and increases reactive oxygen species (ROS) generation, resulting in oxidative damage to cellular macromolecules and disruption of membrane integrity [17]. Similarly, galactose-induced AGEs can exert adverse effects primarily through their interaction with receptors of advanced glycation end products (RAGE), thereby activating nuclear kappa B (NF-κB) signaling and prompting inflammation and oxidative stress [18]. Also, AGE/RAGE signaling promotes events that impair insulin secretion and β-cell toxicity and alter the expression of genes involved in the progression of type-2 diabetes mellitus [18]. Likewise, high cholesterol and fat consumption lead to metabolic stress like dyslipidemia and have adverse effects on the liver, kidney, and reproductive organs [19,20,21]. A combined exposure to high-cholesterol and high-galactose diets serves as an excellent dietary model to induce metabolic stress and resembles the pathophysiology of human fatty liver, insulin resistance, and obesity [22].

The zebrafish was used as a model organism due to its substantial genomic resemblance to humans [23]. In particular, key enzymes, receptors, and lipoproteins involved in lipid metabolism in zebrafish show high similarity to those in humans. For instance, like humans, zebrafish harbor cholesteryl ester transfer protein (CETP), which facilitates cholesterol transfer between lipoproteins, a protein that is absent in rats [24]. In addition, the small size (<5 cm) and ease of handling of zebrafish facilitate the inclusion of large participant pools within limited space. Also, zebrafish embryos are an excellent model for monitoring developmental rate and morphology because they develop externally and are relatively large in size. Furthermore, the embryos are optically transparent during early developmental stages, thereby facilitating precise microinjection of a compound into them [25]. These characteristics allow a direct comparison of developmental progression and morphological changes, making zebrafish embryos a valuable tool for evaluating the efficacy and potential toxicity of compounds. Due to the aforementioned qualities, zebrafish have attracted substantial interest as a preclinical model for studying various human diseases [26], and results from zebrafish can provide useful information for designing human studies.

## 2. Results

### 2.1. Change in Survivability, and Body and Organ Weight of Zebrafish

**Figure 1:**
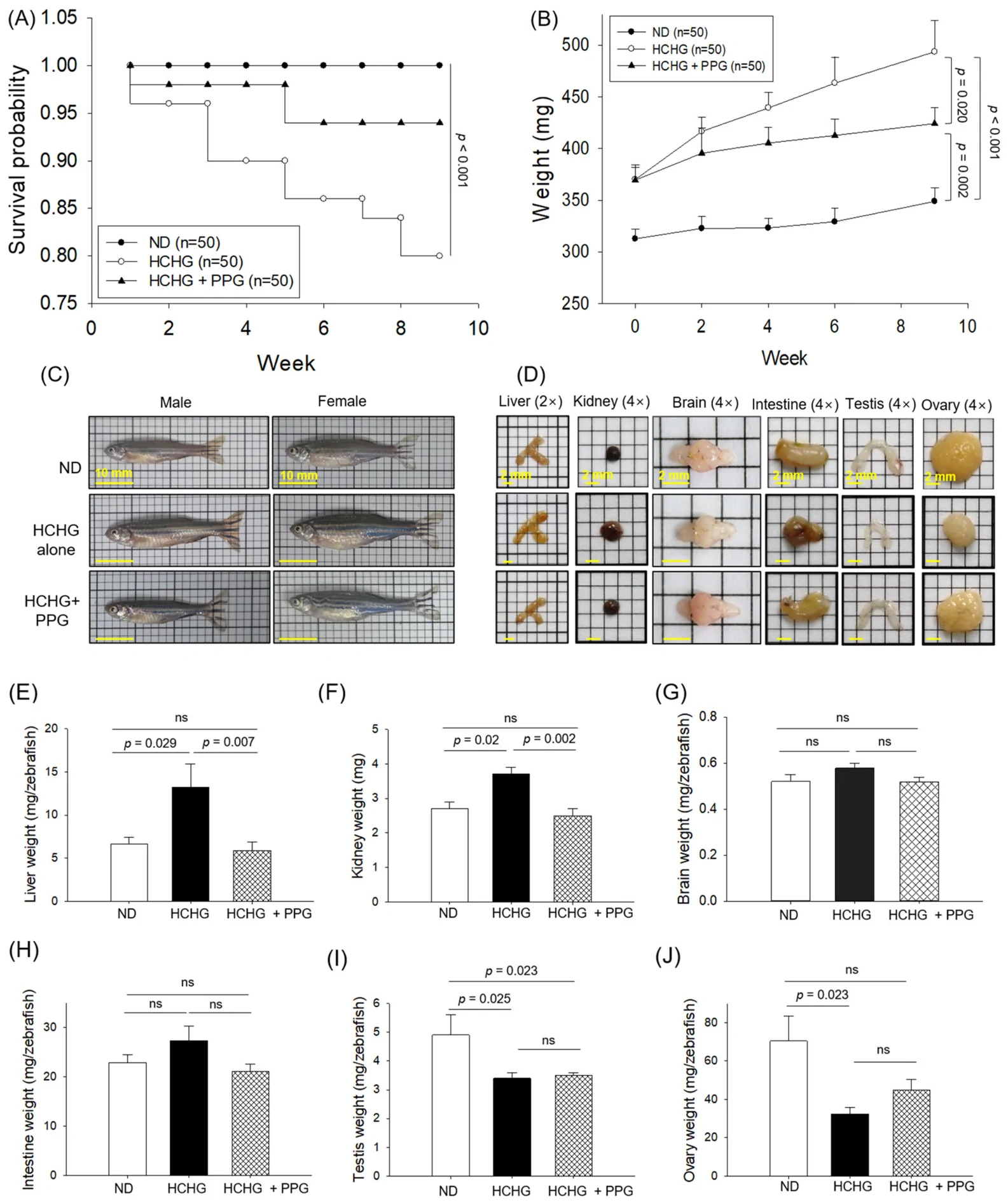
Effect of the mixture of policosanol, phosphatidylserine, and *G. biloba* leaf extract on the hyperlipidemic and hyperglycemic zebrafish during 9 weeks of feeding. (**A**) Kaplan–Meier survival curve of zebrafish. (**B**) Time-dependent (0–9 weeks) body weight change. (**C**) Representative images of male and female zebrafish; scale bar, 10 mm. (**D**) Representative images of various organs, respectively, scale bar, 2 mm. (**E**–**J**) Weight of zebrafish liver, kidney, brain, intestine, testis, and ovary, respectively, after 9 weeks of feeding. ND and HCHG, abbreviated for normal diet and high-cholesterol and high-galactose diet, respectively, whereas PPG represents a mixture of policosanol, phosphatidylserine, and *G. biloba* leaf extract. The *p*-value indicates the statistical significance between the groups evaluated by one-way ANOVA, followed by Tukey’s post hoc analysis; ns indicates a non-significant difference between the groups.

The Kaplan–Meier survival probability curve as depicted in Figure 1A revealed a substantial difference (log-rank *χ*^2^ = 13.4, *p* < 0.001) in survival of zebrafish across the group. In the ND (control) group, the highest absolute zebrafish survival probability (1.0) was observed throughout the 9 weeks of feeding. In contrast, the survival probability of zebrafish in the HCHG group declined severely in a time-dependent manner (Figure 1A). After 2 weeks of HCHG feeding, zebrafish survival probability reached 0.96, then declined to 0.9 after 4 weeks and to 0.8 after 9 weeks. However, when HCHG was co-consumed with PPG, zebrafish survivability notably enhanced (Figure 1A).

A least body weight enhancement with time was observed in the ND (control) group. Compared to week 0 body weight, an 11.6% enhancement in body weight was observed in the ND group after 9 weeks of feeding (Figure 1B). Unlike the ND-fed group, the maximum time-dependent enhancement in body weight was observed in the HCHG group (Figure 1B,C). After week 9 of HCHG feeding, the average zebrafish body weight was 33.4% higher than the body weight observed at week 0. The PPG supplementation proved effective in reducing the HCHG-elevated body weight. Compared to the 33.4% body weight increase in the HCHG group after 9 weeks, PPG supplementation (for 9 weeks) limited the increase to only 14.8% relative to week 0.

The whole-organ analysis revealed hepatic and nephromegaly in the HCHG group, with significant 2-fold (*p* = 0.029) and 1.4-fold (*p* = 0.02) elevations in liver and kidney weight compared to the respective weights observed in the ND (control) group (Figure 1D–F). The HCHG-induced hepatic and nephromegaly was effectively prevented by the co-supplementation of PPG, indicated by significant 2.2-fold (*p* = 0.007) and 1.5-fold (*p* = 0.002) reductions in liver and kidney weight (Figure 1D–F). A non-significant change in the brain and intestine weight was observed across the groups (Figure 1D,G,H). However, the testis and ovary organ morphology and weights were substantially altered by the HCHG, as reflected by a significant 1.4-fold (*p* = 0.025)- and 2.3-fold (*p* = 0.023)-lower testis and ovary weight in the HCHG-fed group compared to the ND control group (Figure 1D,I,J). No significant (*p* > 0.05) effect of PPG supplementation was observed on the HCHG-induced weight changes in the testis and ovary.

### 2.2. Change in Blood Lipid and Glucose Profile

**Figure 2:**
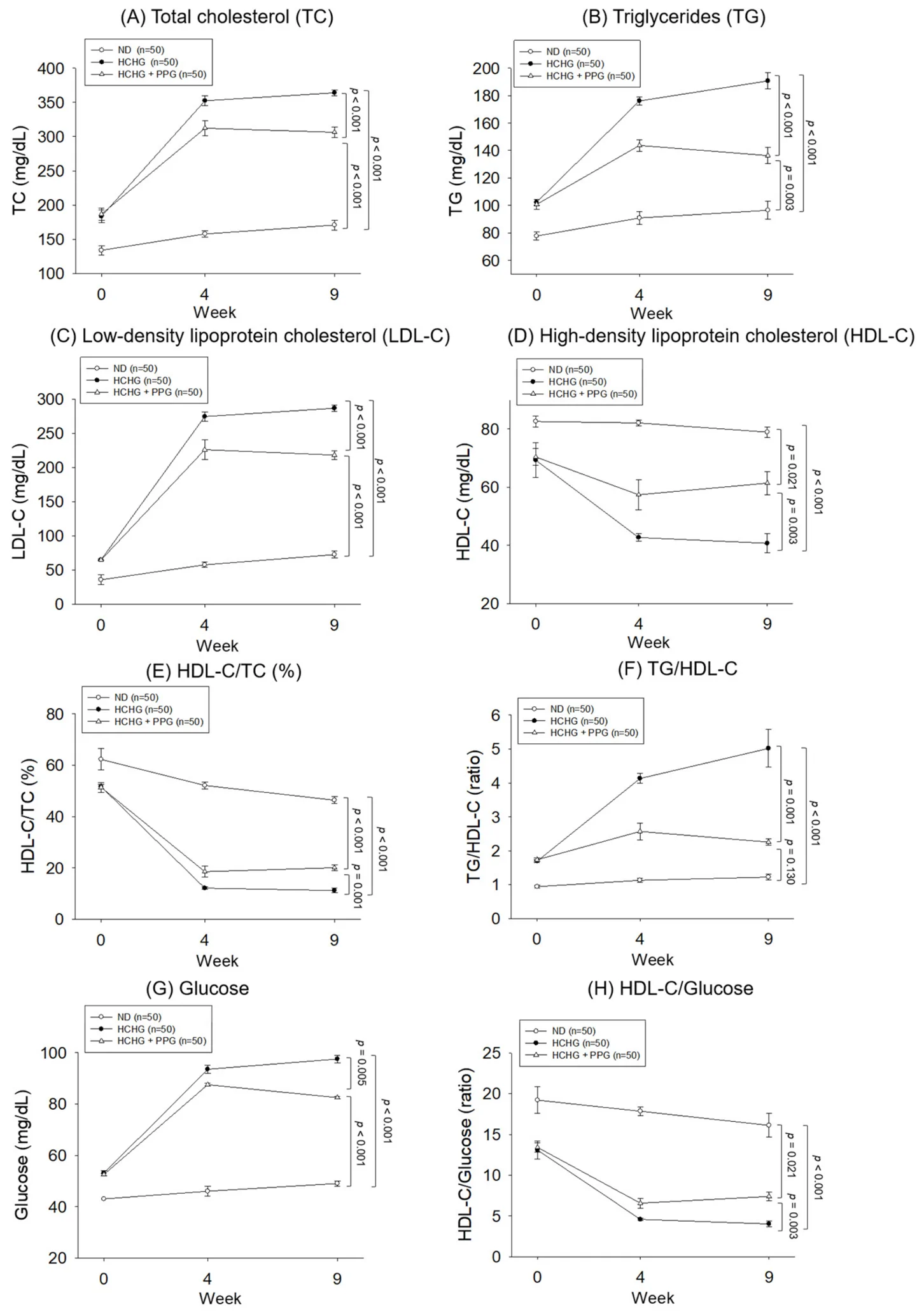
Plasma lipid profile (**A**–**F**), glucose level (**G**), and HDL-C/glucose (**H**) ratio in hyperlipidemic and hyperglycemic zebrafish following 9 weeks of supplementation with the mixture of policosanol, phosphatidylserine, and *G. biloba* leaf. ND and HCHG represent a normal diet and a high-cholesterol and high-galactose diet, respectively, whereas PPG represents a mixture of policosanol, phosphatidylserine, and *G. biloba* leaf extract. Each point in the line graph represents the men ± SEM value obtained from five (*n* = 5) independent experiments. The *p*-value indicates the statistical significance between the groups evaluated by one-way ANOVA, followed by Tukey’s post hoc analysis.

The blood lipid profile of the HCHG-consumed group revealed a time-dependent enhancement in TC and TG levels in the HCHG-supplemented groups, which were significantly higher than the respective levels detected in the ND-supplemented group (Figure 2A,B). Compared to the week 0 value (183.5 mg/dL), the TC level in the HCHG group was enhanced to 352.4 mg/dL after 4 weeks of intake of HCHG, which was further enhanced slightly (363.9 mg/dL) with the feeding time up to 9 weeks, representing a 1.9-fold enhancement in the TC level compared to the week 0 value. Compared with the HCHG group, the HCHG co-supplemented with PPG group showed a lower enhancement in TC level (312.2 mg/dL) after 4 weeks, which then slightly declined (306.3 mg/dL) with increasing feeding time up to 9 weeks. Compared with the week 9 TC level in the HCHG group, PPG supplementation resulted in a 1.2-fold (*p* < 0.001)-lower TC level.

In the ND (control) group, post 9 weeks of feeding, a 1.2-fold enhancement in TG level was observed compared to the TG level of week 0. In contrast, a 1.9-fold increase in TG levels was observed in the HCHG group after 9 weeks of feeding compared with the week 0 value (Figure 2B). A significant 1.4-fold (*p* < 0.001)-lower TG level was detected in the PPG-supplemented group than the TG level of the HCHG group after 9 weeks of feeding. Likewise, a significant reduction in LDL-C (*p* < 0.001) was observed in the PPG co-supplement group compared to the HCHG group (Figure 2C).

A time-dependent reduction in HDL-C levels from 69.3 mg/dL to 40.7 mg/dL, was observed in the HCHG group at weeks 0, 4, and 9 (Figure 2D). Compared with the HCHG group, the PPG-supplemented group showed a 1.5-fold-higher (*p* = 0.003) HDL-C level after week 9 of feeding. A similar trend for the HDL-C/TC ratio was observed, where, compared with the HCHG group, the PPG-supplemented group showed a significant 1.8-fold-higher (*p* = 0.001) HDL-C/TC ratio after week 9 of feeding (Figure 2E). Also, compared with the HCHG group, the PPG group showed a significant 2-fold (*p* < 0.003)-reduced TG/HDL-C ratio after 9 weeks of supplementation (Figure 2F).

The blood glucose level was sharply elevated during 9 weeks of supplementation in both the HCHG (97.5 mg/dL) and PPG (82.5 mg/dL) consumption groups relative to the week 0 value (Figure 2G). However, compared with the HCHG group, the PPG group showed a significantly 1.2-fold (*p* < 0.005)-lower blood glucose level after 9 weeks of consumption.

After 9 weeks of feeding, the highest HDL-C/glucose ratio was observed in the ND (control), followed by the PPG-supplemented group, while the lowest was in the HCHG-supplemented group (Figure 2H). Compared to the HCHG-supplemented group, a significantly 1.8-fold-higher (*p* = 0.003) HDL-C/glucose ratio was reported in the PPG-supplemented group.

### 2.3. Change in Antioxidant Status and Hepatic Damage Biomarkers

**Figure 3:**
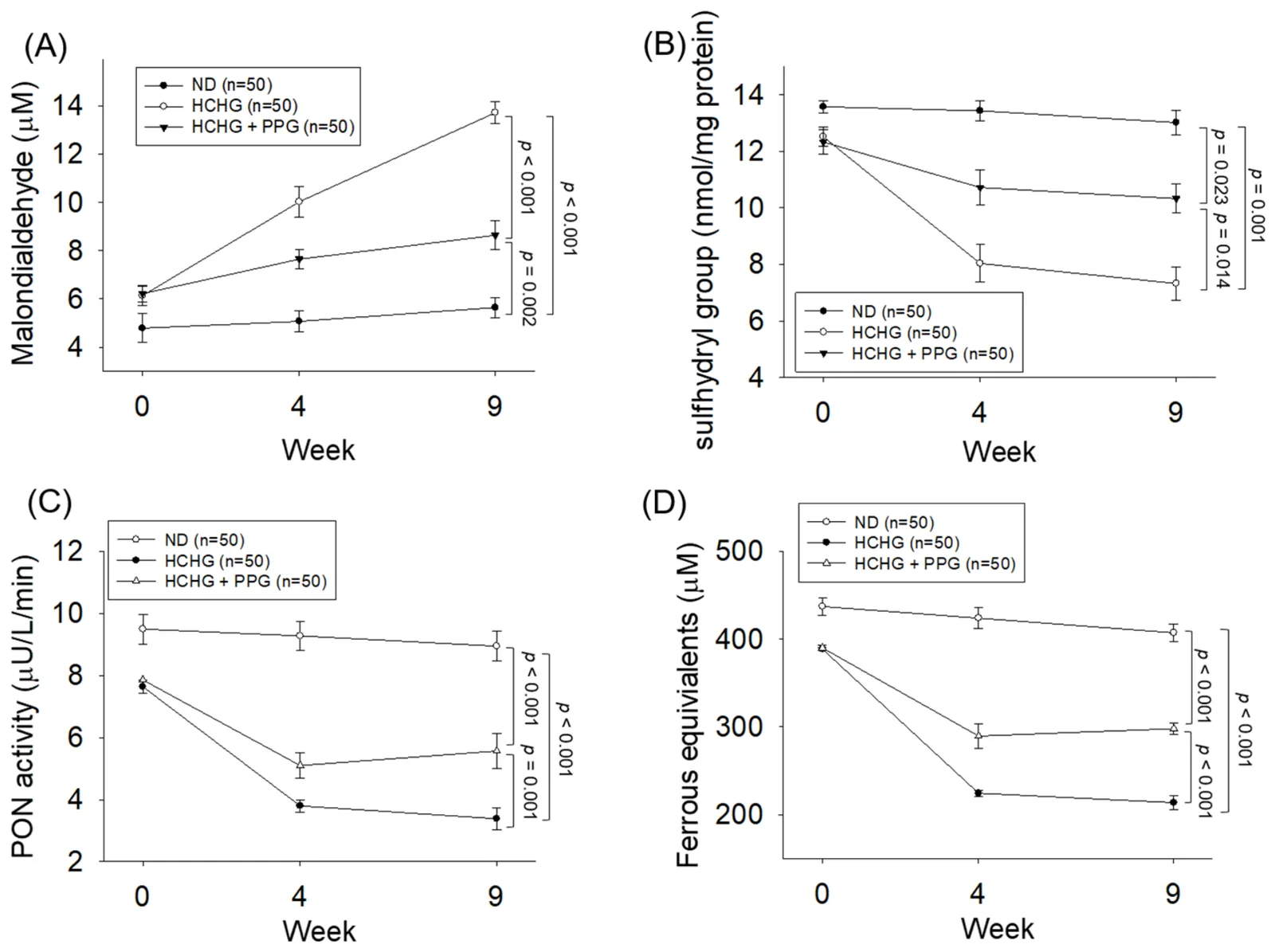
Effect of 9 weeks of feeding a mixture of policosanol, phosphatidylserine, and *G. biloba* leaf extract on zebrafish plasma antioxidant and oxidative stress variables. Plasma levels of (**A**) malondialdehyde (MDA), (**B**) sulfhydryl contents, (**C**) paraoxonase (PON)-like activity, and (**D**) ferric ion reduction ability (FRA). ND and HCHG, abbreviated for normal diet and high-cholesterol and high-galactose diet, respectively, whereas PPG represents a mixture of policosanol, phosphatidylserine, and *G. biloba* leaf extract. Each point in the line graph represents the mean ± SEM value obtained from five (*n* = 5) independent experiments. The *p*-value indicates the statistical significance between the groups evaluated by one-way ANOVA, followed by Tukey’s post hoc analysis.

Compared to the ND (control) group, a significant time-dependent linear enhancement in the plasma MDA level from week 0 (5.2 μM), week 4 (10.1 μM), to week 9 (13.6 μM) was observed in the HCHG-fed group (Figure 3A). Compared with week 0, MDA levels increased 2.6-fold after 9 weeks in the HCHG-consuming group. In contrast, only a 1.5-fold (*p* < 0.001) increase in MDA levels was observed in the PPG co-supplemented group after 9 weeks (8.2 μM), relative to the week 0 value (5.4 μM).

In the ND control group, a nearly constant sulfhydryl content was observed from week 0 to week 9 of feeding. Contrary to this, a sharp decline in sulfhydryl content from 12.9 mmol/mg (week 0) to 7.5 mmol/mg (week 9), accounting for a 1.7-fold reduction, was observed in the HCHG group (Figure 3B). In contrast, only a 1.2-fold reduction in sulfhydryl contents from week 0 (12.8 mmol/mg) to week 9 (10.5 mmol/mg) was observed in the PPG-supplemented groups.

Nearly static plasma PON and FRA activities were observed in the ND (control) group during weeks 0 to 9 of feeding. In contrast, the plasma PON and FRA activities declined by 2.2- and 1.8-fold after 9 weeks of consumption of HCHG compared to the respective activities observed at week 0 (Figure 3C,D). Nevertheless, only 1.4- and 1.3-fold reductions in PON and FRA activities were observed in the PPG group after 9 weeks compared with week 0 values.

### 2.4. Hepatic Damage Biomarkers

**Figure 4:**
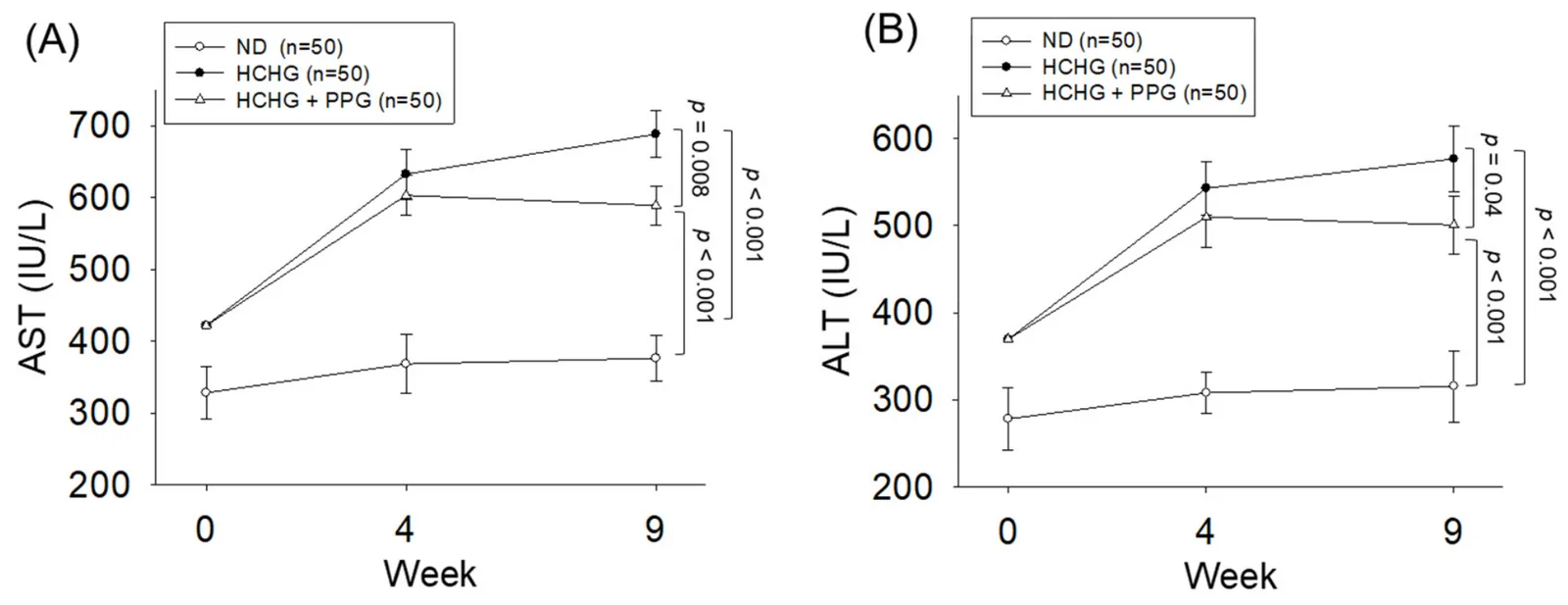
Effect of 9 weeks of feeding a mixture of policosanol, phosphatidylserine, and *G. biloba* leaf extract on zebrafish plasma hepatic function markers (**A**) aspartate aminotransferase (AST) and (**B**) alanine aminotransferase (ALT) activity. ND and HCHG, abbreviated for normal diet and high-cholesterol and high-galactose diet, respectively, whereas PPG represents a mixture of policosanol, phosphatidylserine, and *G. biloba* leaf extract. Each point in the line graph represents the mean ± SEM value obtained from five (*n* = 5) independent experiments. The *p*-value indicates the statistical significance between the groups evaluated by one-way ANOVA, followed by Tukey’s post hoc analysis.

No substantial change in the plasma AST and ALT levels from week 0 to week 9 was observed in the ND (control) group. (Figure 4A,B). Compared to the ND (control) group, ~1.8-fold (*p* < 0.001)-higher AST and ALT levels were detected in the HCHG group after 9 weeks of feeding. The co-consumption of PPG resulted in a ~1.2-fold reduction in AST (*p* < 0.008) and ALT (*p* < 0.004) levels compared to the HCHG group after 9 weeks of feeding, underscoring PPG inhibitory effect on HCHG-induced elevations in AST and ALT.

### 2.5. Microinjection of Plasma into Zebrafish Embryos

**Figure 5:**
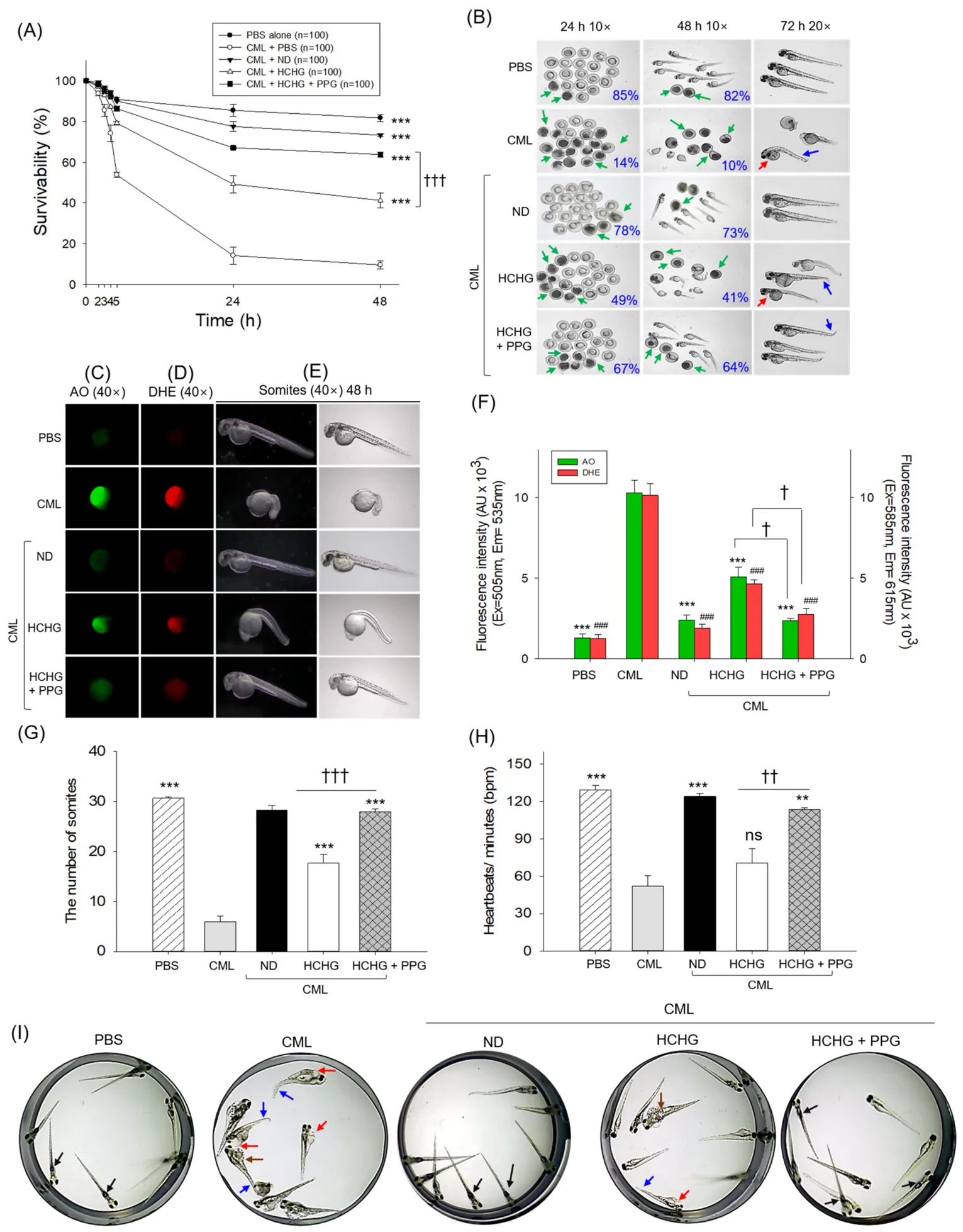
Effect of plasma obtained from the zebrafish following feeding of a mixture of policosanol, phosphatidylserine, and *G. biloba* leaf extract on the carboxymethyllysine (CML)-induced toxicity in zebrafish embryos. (**A**) Kinetics of embryo survivability during 48 h post-treatment. (**B**) Representative images depicting morphological changes. Green arrows represent dead embryos; red and blue arrows highlight the pericardial edema and tail fin curvature, respectively. (**C**,**D**) Dihydroethidium (DHE), acridine orange (AO) fluorescent staining. (**E**) Images depicting somites in embryos at 48 h post-treatment. (**F**) Quantification of the DHE and AO-fluorescent intensity. (**G**,**H**) Quantification of somite numbers and heartbeat. (**I**) Developed embryo’s morphology at 144 h post-treatment. Black arrows represent inflated swimming bladder; red, blue and brown arrows highlight the pericardial edema, tail fin curvature and yolk sac edema, respectively. ND and HCHG, abbreviated for normal diet and high-cholesterol and high-galactose diet, respectively, whereas PPG represents a mixture of policosanol, phosphatidylserine, and *G. biloba* leaf extract. ** (*p* < 0.01) and *** (*p* < 0.001) represent the statistical difference compared to the CML-injected groups, ^###^ (*p* < 0.001) highlights the statistical difference for DHE fluorescent intensity compared to the CML-injected group, whereas ^†^ (*p* < 0.05), ^††^ (*p* < 0.01) and ^†††^ (*p* < 0.001) indicate statistically significant differences between the marked groups using one-way ANOVA, following Tukey’s post hoc analysis; ns indicates a non-significant difference between the groups.

The plasma functionality was assessed by testing its efficacy against CML-induced oxidative insult in zebrafish embryos. In response to CML, the survivability of embryos severely declined in a time-dependent manner and finally reached 10% at 48 h post-treatment. In contrast, the highest embryo survival (83.3%) was observed in the PBS-injected (control) group. The CML-induced adverse effect on embryo survivability was significantly mitigated by treatment with plasma from the ND-, HCHG- and HCHG+PPG-supplemented groups (Figure 5A,B). However, among the three different plasma samples, the plasma from the ND group showed the highest protective effect in preventing CML-compromised survivability, while the least effect was noticed for the plasma from the HCHG group. Compared to the plasma from the HCHG group, plasma from the PPG-supplemented group showed significantly (*p* < 0.001) higher survivability, as reflected by 65% embryo survivability compared to 45% survivability in the HCHG plasma-treated group at 48 h post-treatment.

Furthermore, a high prevalence of ROS generation and apoptosis was observed in CML-treated embryos, which was significantly reduced by plasma treatment (from the ND-, HCHG- and PPG-supplemented groups) (Figure 5C,D,F). The plasma from the ND-supplemented group showed the highest effect in reducing CML-induced ROS generation and apoptosis. However, compared to the HCHG-treated group, embryos injected with plasma from the PPG-treated groups showed significantly lower ROS (52.7%; *p* < 0.05) and apoptosis (39.6%; *p* < 0.05), indicating higher functionality of plasma from the PPG-treated group than the HCHG-treated group.

Similarly, the CML-dismissed somite numbers were significantly restored by plasma from the ND-, HCHG-, and PPG-treated groups (Figure 5E,G). Nevertheless, embryos injected with plasma from the PPG group showed a significantly (*p* < 0.001) higher somite count (28) than the HCHG-treated group (17.6). A reduced heartbeat as observed in the CML-treated group (52 bpm) was significantly elevated by the treatment of plasma from the ND- (144 bpm, *p* < 0.001) and PPG-supplemented group (114 bpm, *p* < 0.01) (Figure 5H). Surprisingly, a non-significant effect of plasma from the HCHG group was noticed on the restoration of CML-compromised embryo heartbeats.

The outcomes at 72 h and 144 h post-treatment revealed that most surviving embryos (~70%) in the CML-treated group exhibited embryonic developmental deformities related to tailfin curvature, pericardial edema, yolk sac edema, and stunted growth (Figure 5B,I and Supplementary Figures S1 and S2). In contrast, the embryo’s developmental deformities were substantially mitigated by exposure to plasma from ND, HCHG- and PPG-fed groups. However, compared to the HCHG group, reduced developmental deformities were observed in the embryos injected with plasma from ND- and PPG-supplemented groups. Importantly, most embryos from the CML-treated groups (at 144 h) displayed an uninflated swimming bladder, with altered stationary posture and swinging activity (Figure 5I). In contrast, embryos that received plasma from ND-supplemented groups showed an inflated swimming bladder (indicated by a black arrow), stationary posture, and normal swimming activity. Unlike the ND group, most embryos from the HCHG plasma supplemented group showed an uninflated swimming bladder and exhibited impaired swimming activity. In contrast, the majority of embryos treated with plasma from the HCHG co-supplemented with PPG group showed an inflated swimming bladder, stationary posture, and normal swimming activity (Figure 5I and Supplementary Figure S2).

### 2.6. Inflammatory Biomarkers and Senescence in Liver

**Figure 6:**
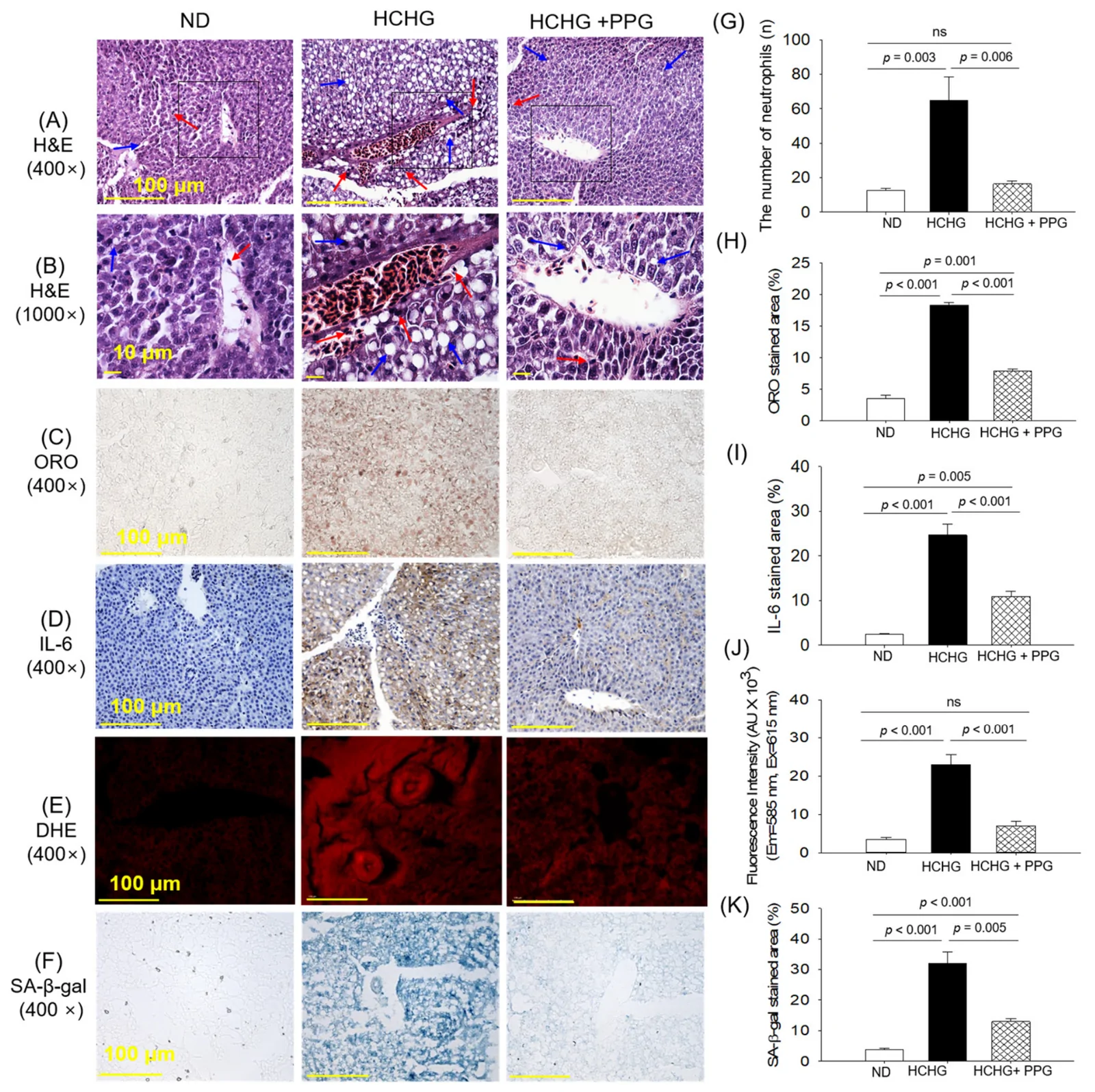
Effect of the mixture of policosanol, phosphatidylserine, and *G. biloba* leaf extract on the hyperlipidemic and hyperglycemic zebrafish liver histology after 9 weeks of feeding. (**A**,**B**) Hematoxylin and eosin (H&E) staining images captured at 400× and 1000× magnification, respectively. Blue and red arrows highlight the lipid droplets and neutrophils, respectively. (**C**) Oil red O staining (ORO). (**D**) Immunohistology (IHC) for the detection of interleukin (IL)-6. (**E**,**F**) dihydroethidium (DHE) fluorescent staining and senescent-associated β-galactosidase (SA-β-gal) staining. Scale bar: 100 μm. Quantification of (**G**) neutrophil infiltration, (**H**) ORO-stained area, (**I**) IL-6-stained, (**J**) DHE fluorescent staining and (**K**) senescence-positive cells. ND and HCHG, abbreviated for normal diet and high-cholesterol and high-galactose diet, respectively, whereas PPG represents a mixture of policosanol, phosphatidylserine, and *G. biloba* leaf extract. ImageJ software (version 1.54s, https://imagej.net/ij; assessed in 16 April 2026) was used to quantify ORO, IL-6, and SA-β-gal staining and DHE fluorescence intensity. For the quantitative analysis, five sections and five random fields per section were analyzed. The *p*-value indicates the statistical significance of the groups determined by one-way ANOVA, following Tukey’s post hoc analysis; ns indicates a non-significant difference between the groups.

The H&E and ORO staining of the liver suggests higher neutrophil infiltration (5.1-fold, *p* = 0.003) and lipid accumulation (5.2-fold, *p* < 0.001) in the HCHG-fed group than in the ND (control) group (Figure 6A–C,G,H). However, compared to the HCHG-fed group, a significant 3.9-fold (*p* = 0.006)- and 2.3-fold (*p* < 0.001)-lower neutrophil count and ORO-stained area were quantified in the PPG co-supplemented group. Likewise, IL-6 production, ROS generation, and senescent-positive cells were significantly reduced by 2.3-fold (*p* < 0.001), 1.6-fold (*p* < 0.001), and 2.8-fold (*p* = 0.005), respectively, in the liver section of the PPG-supplemented group compared with the HCHG-supplemented group (Figure 6D–F,I–K), indicating the effect of PPG on the HCHG-induced changes in the liver.

### 2.7. Renal Morphology, Reactive Oxygen Species (ROS) and Senescence

**Figure 7:**
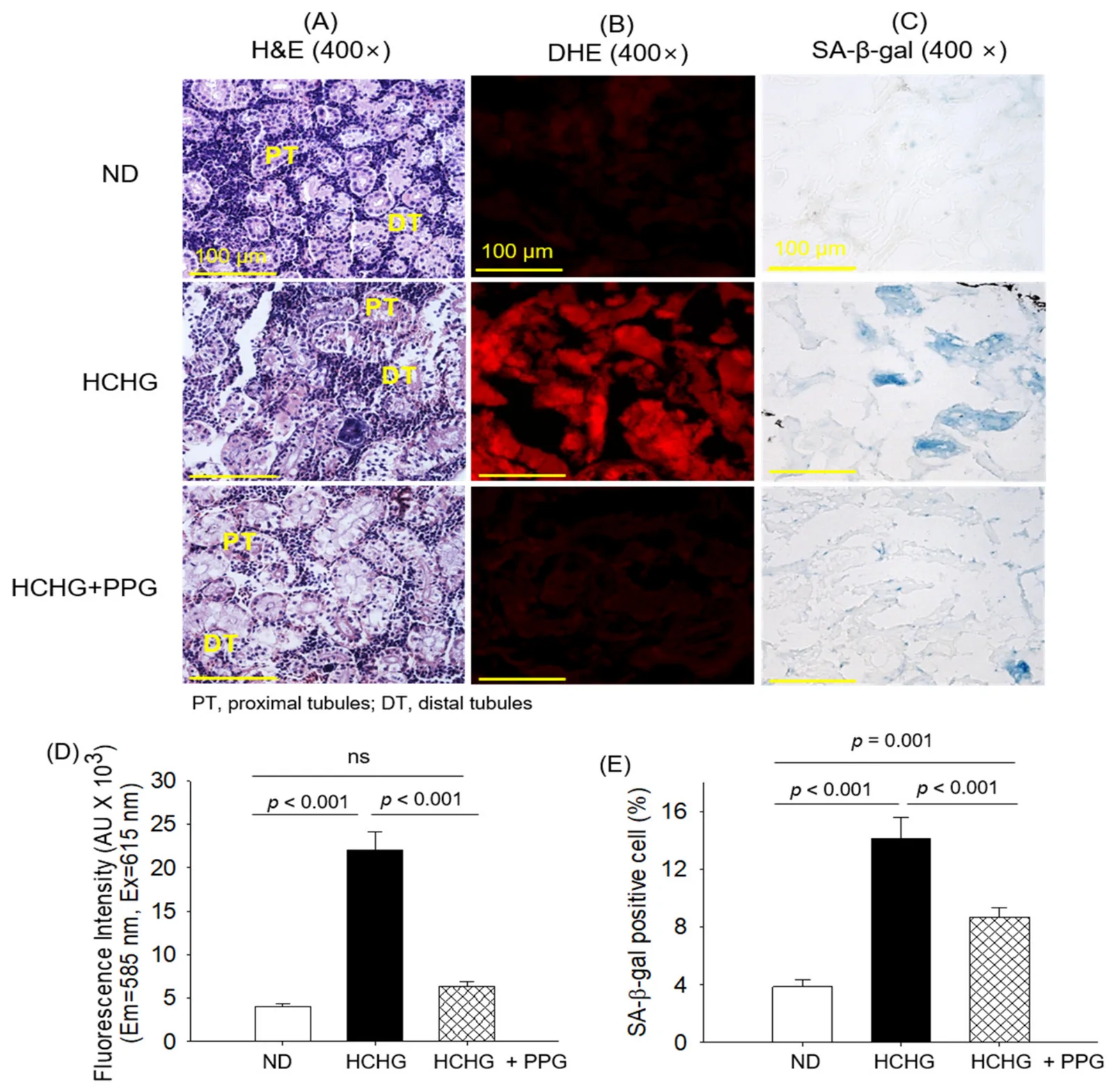
Effect of the mixture of policosanol, phosphatidylserine, and *G. biloba* leaf extract on the hyperlipidemic and hyperglycemic zebrafish kidney histology after 9 weeks of feeding. (**A**) Hematoxylin and eosin (H&E) staining. (**B**,**C**) Dihydroethidium (DHE) fluorescent staining and senescence-associated β-galactosidase (β-gal) staining. Scale bar: 100 μm. Quantification of (**D**) DHE fluorescent staining and (**E**) senescence-positive cells. ND and HCHG, abbreviated for normal diet and high-cholesterol and high-galactose diet, respectively, whereas PPG represents a mixture of policosanol, phosphatidylserine, and *G. biloba* leaf extract. For the quantitative analysis, five sections and five random fields per section were analyzed. The *p*-value indicates the statistical significance of the groups determined by one-way ANOVA, following Tukey’s post hoc analysis; ns indicates a non-significant difference between the groups.

Compared to the ND (control) group, the H&E staining of the kidney revealed distorted and sparsely populated distal (DT) and proximal tubular (PT) structures, along with the presence of cellular debris in the lumen and dilated tubular lumen in the HCHG-fed group (Figure 7A). The co-supplementation of PPG effectively prevented the HCHG-caused morphological changes in the kidney; however, the presence of dilated tubular lumen was observed occasionally. In addition, the DHE and SA-β-gal staining showed a high prevalence of ROS and cellular senescence in the kidney of the HCHG-fed group, which was significantly 5.5-fold (*p* < 0.001)- and 3.7-fold (*p* < 0.001)-higher than the respective levels quantified in the ND (control) group. The PPG co-supplementation effectively reduced ROS and cellular senescence by 3.5-fold (*p* < 0.001) and 1.6-fold (*p* < 0.001), compared to the HCHG group (Figure 7B–E).

### 2.8. Testicular Morphology and Senescence

**Figure 8:**
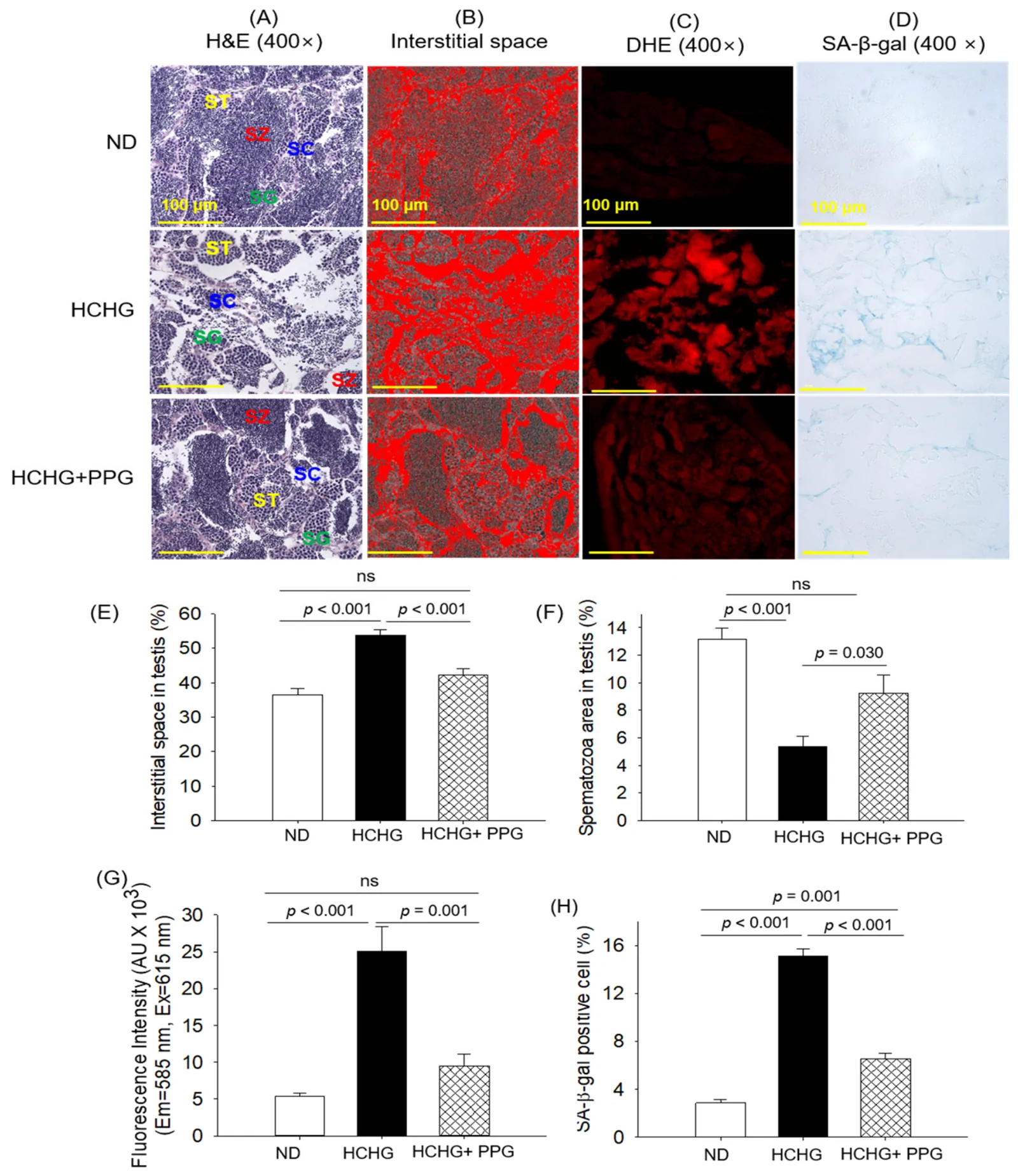
Histological analysis of zebrafish testis post 9 weeks of supplementation with the mixture of policosanol, phosphatidylserine, and *G. biloba* leaf extract. (**A**) Hematoxylin and eosin (H&E) staining. (**B**) Interstitial space between the seminiferous tubules. The interstitial space was determined by performing red conversion in ImageJ (version 1.54s, https://imagej.net/ij; assessed on 16 April 2026) using a 0–120 white color threshold. (**C**) Dihydroethidium (DHE) fluorescent staining and (**D**) senescent-associated β-galactose (SA-β-gal) staining. Scale bar: 100 μm. Quantification of (**E**) interstitial space, and (**F**) spermatozoa area. (**G**,**H**) Quantification of DHE fluorescent intensity and SA-β-gal positive cells. ND and HCHG, abbreviated for normal diet and high-cholesterol and high-galactose diet, respectively, whereas PPG represents a mixture of policosanol, phosphatidylserine, and *G. biloba* leaf extract. For the quantitative analysis, five sections and five random fields per section were analyzed. The *p*-value indicates the statistical significance of the groups determined by one-way ANOVA, following Tukey’s post hoc analysis; ns indicates a non-significant difference between the groups.

The histology of the testis revealed a 1.5-fold (*p* < 0.001)-higher interstitial space between the seminiferous tubules and 2.5-fold (*p* < 0.001)-reduced spermatozoa counts in the testis of the HCHG-supplemented group compared to the ND (control) group (Figure 8A,B,E,F). Compared with the HCHG group, a significantly 1.3-fold (*p* < 0.001)-lower and 1.8-fold (*p* = 0.03)-higher interstitial space and spermatozoa count were detected in the testes of the PPG co-supplemented group. The DHE and SA-β-gal staining revealed a 2.7-fold (*p =* 0.001)- and 2.2-fold (*p* < 0.001)-higher presence of ROS and senescent-positive cells in the HCHG-consumed group compared to the PPG co-supplemented group (Figure 8C,D,G,H). The collective findings suggest a significant effect of PPG on the HCHG-induced damage to the testis.

### 2.9. Ovarian Morphology and Senescence

**Figure 9:**
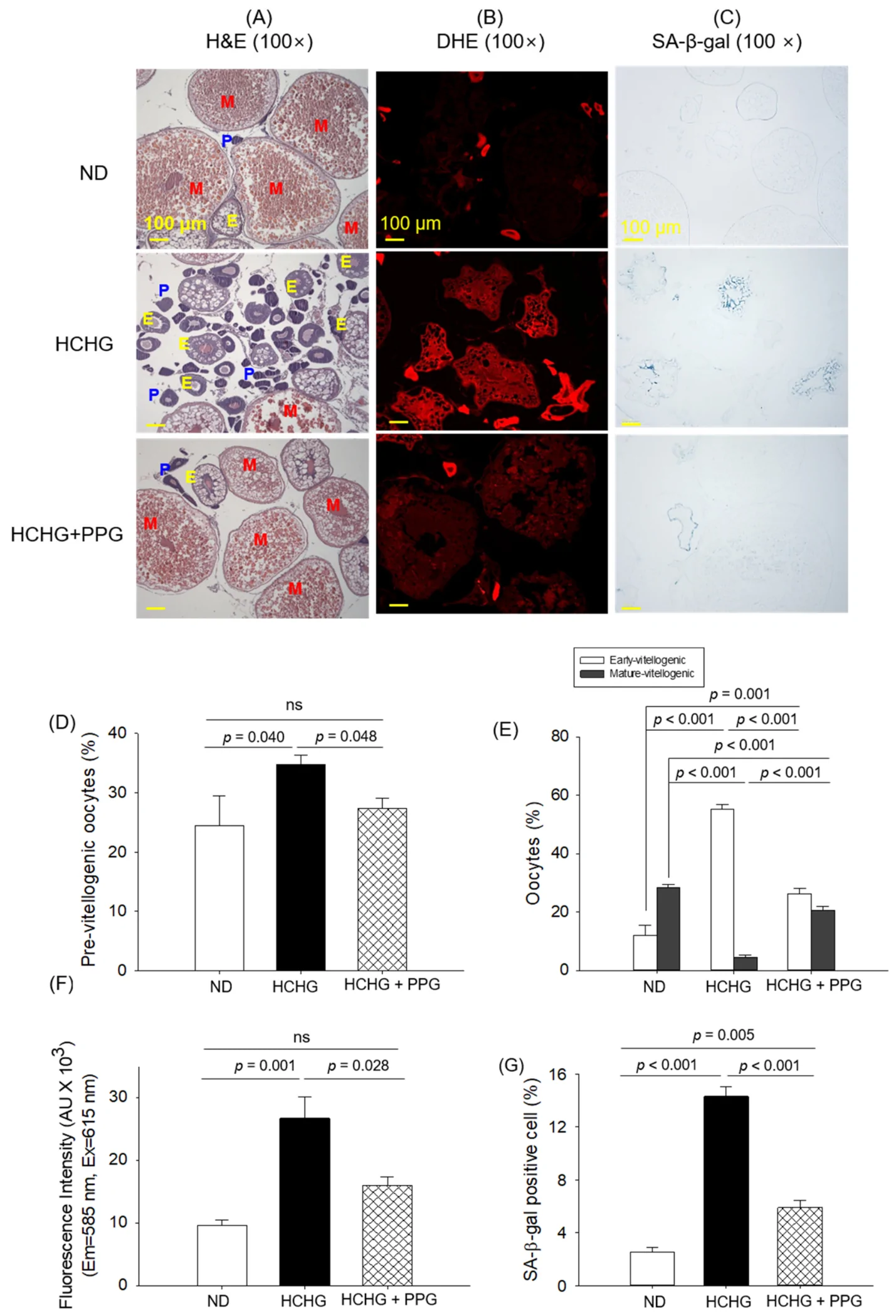
Effect of the mixture of policosanol, phosphatidylserine, and *G. biloba* leaf extract on the hyperlipidemic and hyperglycemic zebrafish ovary histology after 9 weeks of feeding. (**A**) Hematoxylin and eosin (H&E) staining. P, E, and M highlight pre-, early-, and mature-vitellogenic oocytes, respectively. (**B**) Oil red O staining (ORO). (**C**) Senescence-associated β-galactosidase (SA-β-gal) staining. Quantification of (**D**) pre-vitellogenic oocytes, (**E**) early and mature oocytes, (**F**) DHE fluorescent intensity, and (**G**) senescence (SA-β-gal) positive cells. ND and HCHG, abbreviated for normal diet and high-cholesterol and high-galactose diet, respectively, whereas PPG represents a mixture of policosanol, phosphatidylserine, and *G. biloba* leaf extract. For the quantitative analysis, five sections and five random fields per section were analyzed. The *p*-value indicates the statistical significance of the groups determined by one-way ANOVA, following Tukey’s post hoc analysis; ns indicates a non-significant difference between the groups.

The H&E staining of the ovary depicts a high number of pre-vitellogenic (34.8%) and early vitellogenic (27.4%) oocytes, while a low prevalence of mature oocytes (10.1%) was observed in the HCHG-supplemented group, which significantly differed from the ND (control) group (Figure 9A,D,E). Co-supplementation with PPG significantly reversed the HCHG-induced alteration in oocyte counts. As depicted in Figure 9, a significant 1.2-fold (*p* = 0.048)- and 2.1-fold (*p* < 0.001)-lower number of pre- and early vitellogenic oocytes and 4.6-fold (*p* < 0.001)-higher mature oocytes were observed in the PPG-supplemented group compared to the HCHG group.

The DHE and SA-β-gal staining revealed a 2.8-fold (*p* = 0.001)- and 5.7-fold (*p* < 0.001)-higher ROS production and senescence in the ovaries of the HCHG group, compared to the ND (control) group. The co-supplementation of PPG significantly reduced the HCHG-elevated ROS and senescence by 2.6-fold (*p* = 0.028) and 7.4-fold (*p* < 0.001) (Figure 9B,C,F,G).

### 2.10. Intestinal Morphology and Collagenation

**Figure 10:**
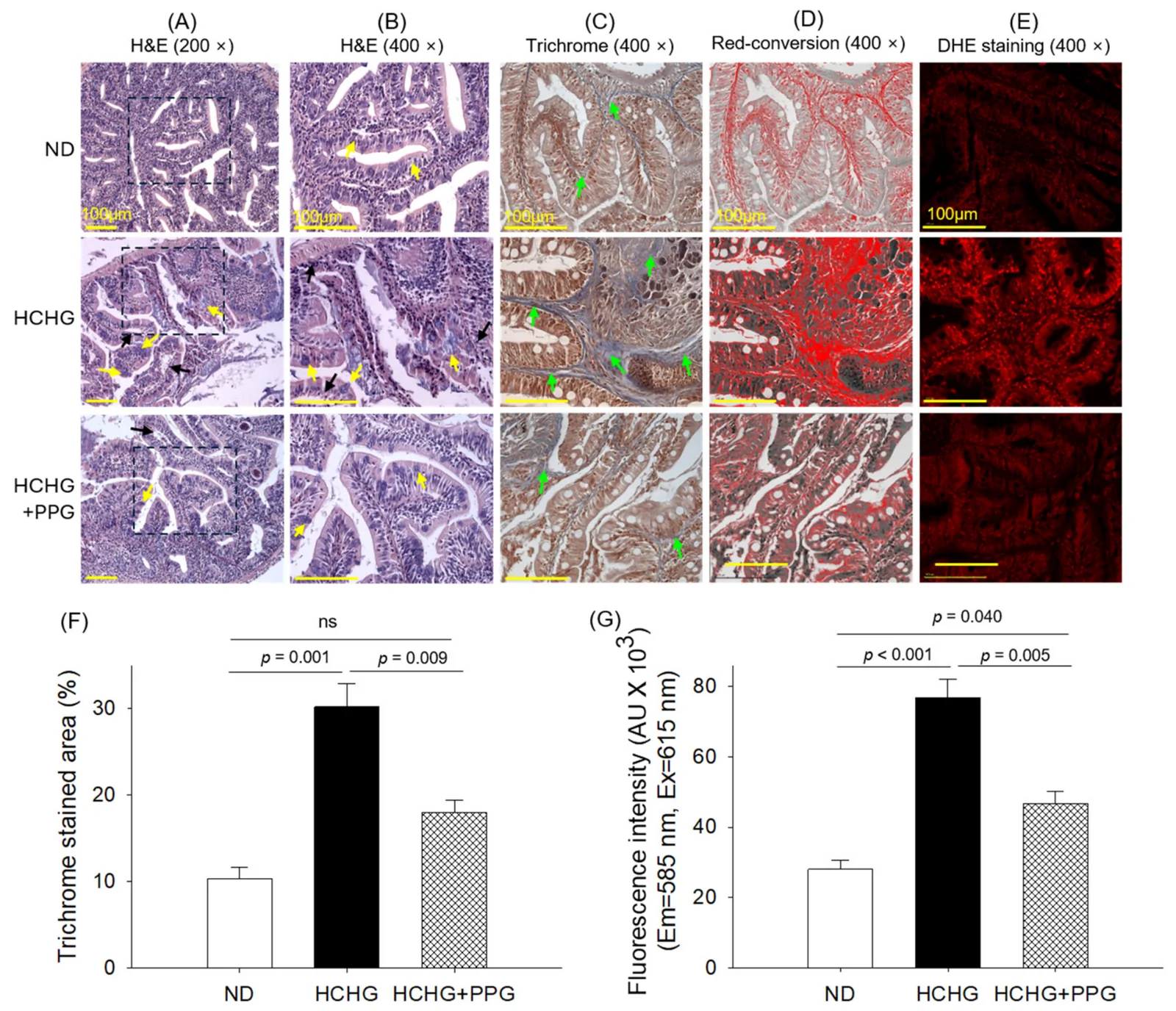
Histological analysis of the zebrafish intestine after 9 weeks of supplementation with a mixture of policosanol, phosphatidylserine, and *G. biloba* leaf extract. (**A**,**B**) Hematoxylin and eosin (H&E) staining at 200× and 400× magnification, respectively. Black and yellow arrows highlight degeneration of the lamina propria and hypertrophy of goblet cells. (**C**) Masson-trichrome staining. Green arrow highlights a collagenated area. (**D**) The Masson-trichrome-stained blue area was replaced with red to enhance visibility. The red conversion was performed at the blue color threshold of 20–120 using ImageJ software (version 1.54s, https://imagej.net/ij; assessed in 16 April 2026). (**E**) Dihydroethidium (DHE) fluorescent staining. Quantification of (**F**) Masson-trichrome-stained area and (**G**) DHE fluorescent intensity. ND and HCHG, abbreviated for normal diet and high-cholesterol and high-galactose diet, respectively, whereas PPG represents a mixture of policosanol, phosphatidylserine, and *G. biloba* leaf extract. For the quantitative analysis, five sections and five random fields per section were analyzed. The *p*-value indicates the statistical significance of the groups determined by one-way ANOVA, following Tukey’s post hoc analysis; ns indicates a non-significant difference between the groups.

In contrast to the ND (control) group, the H&E staining of the intestine from the HCHG-supplemented group showed a disruption of villus structure with degeneration of the lamina propria (indicated by a black arrow) and hypertrophy of goblet cells (indicated by a yellow arrow) (Figure 10A,B). The HCHG-induced histological changes were substantially minimized by co-supplementation with PPG. However, occasional degeneration of the lamina propria and goblet cell hypertrophy were observed at certain locations in the PPG-supplemented groups.

Masson-trichrome staining (Figure 10C,D,F), demonstrated a significant 2.9-fold (*p* = 0.001) increase in the collagenated area in the intestinal section of the HCHG group compared with the ND group, indicating fibrosis and damage to the inner circular muscle and surrounding connective tissue. The co-supplementation of PPG with HCHG substantially prevented tissue fibrosis, as reflected by a significantly 2.2-fold (*p* = 0.009)-reduced collagenated area in the PPG-supplemented group than that of the HCHG-fed group. Consistently, DHE fluorescence staining revealed high ROS production in the HCHG-supplemented group, which was significantly reduced by 1.8-fold (*p =* 0.005) following co-supplementation with PPG (Figure 10E,G).

### 2.11. Brain Histology

**Figure 11:**
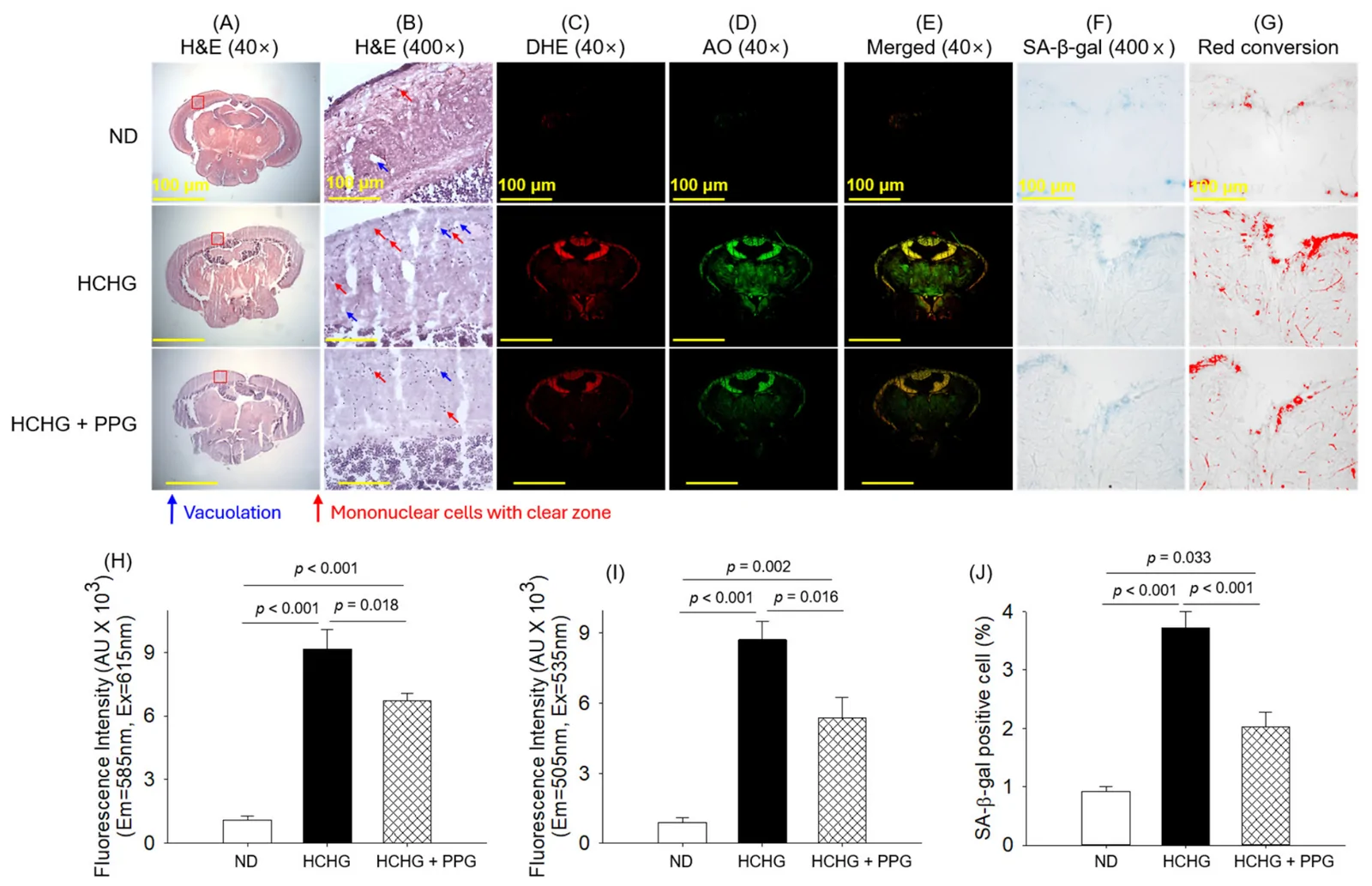
Histological analysis of the zebrafish brain after 9 weeks of supplementation with a mixture of policosanol, phosphatidylserine, and *G. biloba* leaf extract. (**A**,**B**) Hematoxylin and eosin (H&E) staining at 200× and 400× magnification, respectively. (**C**–**E**) Dihydroethidium (DHE), acridine orange (AO) fluorescent intensity, and merged images of DHE- and AO-stained sections, respectively. (**F**) Senescent-associated β-galactosidase (SA-β-gal) staining. (**G**) Red conversion of the senescent-positive blue cells to enhance visibility using ImageJ software (version 1.54s, https://imagej.net/ij; assessed on 16 April 2026) at a blue color threshold value of 0–120. (**H**–**J**) Quantification of DHE, AO and senescent positive cells, respectively. ND and HCHG, abbreviated for normal diet and high-cholesterol and high-galactose diet, respectively, whereas PPG represents a mixture of policosanol, phosphatidylserine, and *G. biloba* leaf extract. For the quantitative analysis five sections and five random fields per section were analyzed. The *p*-value indicates the statistical significance of the groups determined by one-way ANOVA, following Tukey’s post hoc analysis.

As compared to the ND (control) group, the brain histology, as depicted in Figure 11, showed a high presence of mononuclear cells with a clear zone and vacuolation in the tectum opticum (TeO) and the periventricular gray zone (PGZ) of the brain section from the HCHG group compared to the PPG supplement group. A significant 8.3-fold (*p* < 0.001)-higher ROS and 9.5-fold (*p* < 0.001)-higher apoptosis were observed in the TeO and PGZ regions of the HCHG group compared to the ND (control) group, which were significantly reduced by 1.4-fold (*p =* 0.018) and 1.6-fold (*p =* 0.016), respectively, with co-supplementation of PPG (Figure 11C–E,H,I). Also, a 4-fold (*p* < 0.001)-higher prevalence of senescence in the HCHG group compared to the ND group was observed (Figure 11F,G,J). The PPG supplementation effectively reduced the HCHG-elevated senescence by 1.9-fold (*p* < 0.001). The results highlighted the effect of PPG in mitigating HCHG-induced adverse events in the brain.

## 3. Discussion

High cholesterol and high galactose consumption are related to several adverse outcomes, leading to an impact on a variety of organ functions. A combined intake of high cholesterol and galactose served as an excellent dietary model to induce metabolic stress that resembles the physiology of human metabolic disorders [22]. Herein, in response to HCHG consumption, high mortality in zebrafish was observed, which was substantially mitigated by co-supplementation with PPG. Consistently, PPG effectively mitigated the HCHG-triggered elevation in body weight and demonstrated its protective effect against HCHG-induced events. The higher survivability and lower body weight gain in the PPG-supplemented group are attributed to the presence of policosanol, phosphatidylserine, and *G. biloba*, which have been recognized for their anti-obesity effects. In combination, these three components worked efficiently to mitigate HCHG-induced mortality and body weight gain. It has been documented that policosanol enhances energy expenditure in adipose tissue [2] and increases brown adipose tissue activity, leading to weight management in diet-induced obesity [27]. Also, the effect of *G. biloba* in reducing adipocyte volume and adipose tissue lipid accumulation [28], and its inhibitory effect on hepatic pancreatic lipase, have been documented as important anti-obesity effects [29]. Of note, a substantial effect of phosphatidylserine has been recognized in mitigating high-fat diet-induced obesity by targeting the gut microbiota and modulating bile acid metabolism [30]. Owing to the above-reported properties, the presence of these constituents in PPG may work in a complementary manner to effectively mitigate HCHG-induced body weight gain.

A notable effect of PPG was observed in counteracting HCHG-induced dyslipidemia. The presence of policosanol and *G. biloba* in PPG is a major contributor to the counteraction of HCHG-triggered dyslipidemia. Precisely, policosanol has been well described for its potential to lower blood cholesterol levels by targeting HMG-CoA reductase, an important rate-limiting enzyme in cholesterol biosynthesis [2]. In addition, policosanol impacts cholesterol efflux capacity [3] and cholesterol catabolism in the liver by facilitating the conversion of cholesterol to bile acid and its subsequent removal from feces [31], which also contributes significantly to the cholesterol-lowering effect. The notably high levels of HDL-C and HDL-C/TC in the PPG-supplemented group are attributable to the presence of policosanol. An established effect of policosanol has been shown to inhibit the cholesteryl ester transfer protein (CETP) [5], leading to the augmentation of HDL-C blood levels. Moreover, policosanol induces the expression of apolipoprotein A-I (apoA-I), a major HDL-C protein, thereby augmenting HDL-C levels [5]. In addition to policosanol, the effects of *G. biloba* [32,33] and phosphatidylserine [34] on reducing TC and TG and elevating HDL-C levels have been reported. Moreover, in patients with human dyslipidemia, *G. biloba* in combination with statins showed greater efficacy in reducing TC and TG and elevating HDL-C levels compared with statins alone [35]. Also, ginkgolide (B), a purified phytoconstituent from *G. biloba*, showed a substantial effect in reducing high-fat diet-elevated TC and TG levels in rats by activating the peroxisome proliferator-activated receptor alpha (PPARα) signaling pathway [36]. Taken together, the current outcomes, along with the evidence from the literature, suggest that the presence of policosanol, *G. biloba* and phosphatidylserine in PPG may work cooperatively to counteract HCHG-induced dyslipidemia.

Intake of high levels of cholesterol and galactose has been linked to oxidative stress, as reflected by elevated plasma MDA levels and diminished sulfhydryl content, both of which are important oxidative stress markers associated with various disease conditions [37,38]. Also, FRA, which represents the total antioxidant activity of blood [39] and HDL-associated antioxidant paraoxonase (PON) [40] activity, was substantially diminished in the HCHG group, indicating compromised antioxidant status. The HCHG-induced plasma oxidative stress and impaired antioxidant status were substantially reversed by co-supplementation with PPG. The presence of policosanol and *G. biloba* in PPG contributes substantially to mitigating HCHG-triggered oxidative stress and diminished antioxidant levels. The statement is in accordance with earlier reports documenting the cellular antioxidant effects of policosanol [41] and *G. biloba* [42,43], which prevent lipid peroxidation (assessed by MDA analysis) induced by various external stressors. Precisely, policosanol has been widely recognized to reduce plasma MDA levels and augment sulfhydryl content, FRA, and PON activity against external stress [41]. Also, high galactose levels promote the formation of advanced glycation end products (AGEs), which have a variety of deleterious effects, including oxidative stress [44]. The anti-glycation effect of policosanol has been documented, which helps inhibit AGE formation and, consequently, ROS generation [45]. In addition to policosanol, the *G. biloba* antioxidant effect has been documented as a direct free radical scavenger [10] and a positive impact on intrinsic antioxidants such as superoxide dismutase (SOD), catalase, and glutathione peroxidase (GSH-Px) [46]. In addition, studies have documented that *G. biloba* leaf extract can activate the nuclear factor (erythroid-derived 2)-like 2 (Nrf2) [47], an important transcriptional factor that regulates the antioxidant response to mitigate oxidative stress. The combined presence of policosanol and *G. biloba* leaf extract in PPG leads to a reduction in HCHG-induced oxidative stress and an augmentation of antioxidant variables.

CML has been recognized as inducing oxidative stress and inflammation [48], leading to detrimental effects on zebrafish embryos [49]. Zebrafish embryos serve as an ideal experimental model to evaluate the functionality of a substance under external stress [50]. Herein, plasma functionality was examined against CML-induced toxicity in embryos, and the findings indicate that plasma from both HCHG- and PPG-fed zebrafish substantially protects against CML-induced oxidative stress and death. However, a markedly higher plasma functionality was observed in the PPG- compared to the HCHG-supplemented group. Higher functionality correlates with a higher plasma antioxidant status in the PPG group than in the HCHG group. The result aligned with findings on plasma antioxidant status, with higher PON and FRA activities observed in the PPG group than in the HCHG group.

A notable effect of a high-fat diet, glucose and oxidative stress has been described for insulin resistance, and secretion leads to hyperglycemia [51,52]. In the present study, in response to HCHG, blood glucose levels increased and were substantially reduced by PPG intake. The presence of policosanol, an important constituent of PPG, contributed substantially to the glucose-lowering effect. The statement is in accordance with reports documenting policosanol’s glucose-lowering effect on insulin secretion and sensitization via the AMPK/PI3K/AKT signaling pathway [7]. Similarly, *G. biloba* ameliorates insulin resistance [53], thereby affecting blood glucose regulation. However, reports on the blood-glucose-lowering effects of *G. biloba* are inconsistent [54], strengthening the importance of policosanol in PPG as a key regulator of blood glucose levels.

High cholesterol consumption notably causes fatty liver [55], while high galactose supplementation can cause severe oxidative stress and provoke inflammatory cytokine production, including IL-6, IL-10, and TNF-α [56]. Herein, HCHG-induced fatty liver and IL-6 production were substantially prevented by co-supplementation with PPG, documenting PPG’s liver-protective effect. The liver-protective effect of PPG is attributed to the presence of policosanol [45], *G. biloba* [57], and phosphatidylserine [34], which are well reported for their liver-protective activity. The effect of phosphatidylserine has also been documented to prevent hepatic lipid accumulation and to inhibit the transcriptional induction of the proinflammatory IL-6 [34] which also contributed substantially to PPG’s liver-protective effect. Also, lower ROS generation was observed in the PPG co-supplemented with HCHG group compared with the HCHG-alone-supplemented group. The antioxidant properties of policosanol [41,58] and *G. biloba* [46,47] are the reason for the lower ROS in the PPG-supplemented group. Furthermore, the PPG-supplemented group exhibited lower senescence than the HCHG group, likely attributable to reduced ROS levels, given that oxidative stress induced by ROS is a well-established driver of cellular senescence [59]. Consistent with the histological changes, reduced AST and ALT levels, important hepatic function biomarkers [60], were detected in the PPG-supplemented group, confirming the hepatoprotective ability of PPG. High galactose consumption leads to kidney damage via induction of oxidative stress [61] and formation of advanced glycation end products (AGEs) [62]. PPG intake substantially protects against HCHG-induced kidney damage. Also, a substantial effect of PPG appeared to minimize the HCHG-induced ROS production and senescence. The outcomes are supported by earlier reports on the positive effects of policosanol [58] and *G. biloba* [63] on kidney health.

An adverse effect of galactose on brain health, through the impairment of antioxidants and the induction of oxidative stress, has been documented [64]. Galactose accumulation in the brain induces the formation of AGEs, leading to brain damage and cognitive changes [65]. A positive effect of PPG was observed in ameliorating HCHG-induced brain damage by effectively countering oxidative stress, apoptosis, and senescence. In PPG, the specific presence of phosphatidylserine is one of the main ingredients responsible for boosting brain health in the PPG-supplemented group. The statement is based on reports describing an important role of phosphatidylserine in brain-related aging and cognitive impairment [9]. Studies have documented the beneficial effect of phosphatidylserine on brain antioxidants and, consequently, its healing effect on oxidative parameters in the brain [66]. Moreover, the effects of policosanol [58] and *G. biloba* [67] have been documented to protect the brain, neurological disorders, and cognitive impairment. The positive effect of PPG in inhibiting brain oxidative stress leads to events that suppress apoptosis and senescence in the brain, as oxidative stress is directly linked to apoptosis and senescence [68]. In addition to the direct effect of PPG on the brain, enhanced liver and kidney health in the PPG supplement group also contributed significantly to brain protection via the liver–brain [69] and kidney–brain axes [70].

High cholesterol and high galactose consumption cause damage to the reproductive organs [71]. In particular, high galactose levels cause oxidative stress and impair cellular antioxidant defenses, leading to damage to the testis [72] and ovary [73]. Consistently, we observed high ROS generation and senescence in the testis and ovaries in response to HCHG, which were substantially reversed by PPG supplementation. The effects of policosanol [41] and *G. biloba* [74,75] on the protection of reproductive organs have been documented, and their presence in PPG ascertains testis and ovary protection altered by the intake of HCHG. High ROS generation and senescence in response to HCHG were mitigated by PPG intake, suggesting that PPG’s antioxidant action prevents reproductive organ damage. This notion is consistent with earlier reports documenting the protective effects of antioxidants on the testis [72] and ovaries [73] against oxidative insults.

### Limitations and Prospects

Despite the promising effect of PPG supplementation in hyperlipidemic–hyperglycemic zebrafish, the individual effects of policosanol, phosphatidylserine, and *G. biloba* were not evaluated, which represents a major limitation of the present study. Future studies would be designed to compare the individual effects of policosanol, phosphatidylserine and *G. biloba* leaf extract with their combined formulation (i.e., PPG) to determine their relative efficacy in mitigating the HCHG-induced metabolic stress and associated pathological events. Additionally, the effect of plasma from HCHG and HCHG + PPG-supplemented zebrafish would be examined on the development of zebrafish embryos in the absence of any external stress.

## 4. Materials and Methods

### 4.1. Materials

Policosanol (batch no: 310030324) extracted from Cuban sugarcane wax was kindly provided by Raydel Pty. Ltd. (Thornleigh, NSW, Australia). Phosphatidylserine (batch no: 20221204) and *G. biloba* leaf extract (batch no: FN240619-02) were procured from Ven Biotech Pvt. Ltd. (Kanchipuram, Tamil Nadu, India) and Fine Pharma Inc. (Denver, CO, USA), respectively. Detailed certificates of analysis for the used policosanol, phosphatidylserine, and *G. biloba* leaf extract are provided in Supplementary Figures S3–S5. All other chemicals and reagents, unless otherwise stated, were of analytical grade and used as supplied.

### 4.2. Zebrafish Husbandry

Fourteen-week-old zebrafish (wild-type AB strain; *n* = 180) were kept in a tank connected to the circulating water supply (maintained at 28 °C). The water used had a pH of 7.3, chlorine of 0.18 mg/L, turbidity of 16 NTU, dissolved oxygen of 8 mg/mL, total bacterial counts < 100 CFU and was free from pathogenic microorganisms. A certificate of water analysis is provided as Supplementary Figure S6. The zebrafish culture room was equipped with a 14 h light and 10 h dark photoperiod, and zebrafish were maintained in accordance with the standard guidelines of the Committee of Animal Care and Use of Raydel Research Institute (approval no. RRI-24-001, approval date 2 September 2024). Zebrafish were fed a commercial fish diet, normal Tetrabit D49304 (ND, Tetra GmbH, Melle, Germany), twice a day (9 a.m. and 6 p.m.). Before the main experiment, zebrafish were acclimatized for one week under the above-mentioned conditions.

### 4.3. Preparation of Specific Diet

The ND was mixed with 4% (*w*/*w*) cholesterol and 30% (*w*/*w*) galactose to prepare a high-cholesterol, high-galactose diet (HCHG). The HCHG diet was mixed with 0.03% (*w*/*w*) policosanol, 2.5% (*w*/*w*) phosphatidylserine, and 0.9% (*w*/*w*) *G. biloba* leaf extract, and was named the HCHG+PPG diet. To prepare the HCHG diet, 66 g of the normal tetrabit (ND, a commercial zebrafish diet) was supplemented with 4 g of cholesterol and mixed thoroughly with a spatula. Subsequently, chloroform (~50 mL) was added to the mixture (ND+cholesterol) and mechanically agitated for proper distribution of cholesterol. Later, the chloroform was evaporated completely in a fume hood to obtain the high cholesterol (HC) diet. The prepared HC diet was supplemented with 30 g of galactose, mixed properly with a spatula, and then distilled water (~150 mL) was added. The mixture was blended properly with a spatula and left for 4 h at room temperature (RT). Finally, the mixture was re-agitated (~10 min) and processed for freeze-drying to evaporate water. The freeze-dried diet was named the HCHG diet containing 4% (*w*/*w*) cholesterol and 30% (*w*/*w*) galactose. For the preparation of HCHG enriched with policosanol, phosphatidylserine, and *G. biloba* leaf extract, 62.5 g of ND was supplemented with 4 g of cholesterol and mixed thoroughly using a spatula. Subsequently, chloroform (~50 mL) was added to the mixture (ND+cholesterol), and the mixture was mechanically agitated for proper distribution of cholesterol. The chloroform was then completely evaporated in a fume hood to obtain the HC diet. The prepared HC diet was subsequently supplemented with 30 g of galactose, 0.03 g of policosanol, 2.5 g of phosphatidylserine, and 0.9 g of *G. biloba* leaf extract, mixed thoroughly using a spatula, and then distilled water (~150 mL) was added. The mixture was blended properly with a spatula and allowed to stand at RT for 4 h. Finally, the mixture was re-agitated (~10 min) and subjected to freeze-drying to evaporate water. The freeze-dried sample was designated as HCHG enriched with policosanol, phosphatidylserine, and *G. biloba* leaf extract and abbreviated as HCHG+PPG. The prepared diets, HCHG and HCHG+PPG, were stored in a refrigerator (4 °C) for further use. The selected amounts of policosanol, phosphatidylserine and *G. biloba* leaf extract were formulated based on their respective humanized doses recommended by the Ministry of Food and Drug Safety (MFDS), Republic of Korea [1] (Supplementary Figures S7–S9).

Before starting the main feeding experiment, the liking and disliking of zebrafish towards HCHG and HCHG+PPG formulated diets was assessed by the food consumption test using the formula: [total amount of the given food (mg) − remaining amount of food (mg)/Total amount of the given food (mg)] × 100. In both groups, 95–100% food consumption was observed within 15 min of dietary exposure, suggesting zebrafish have a similar preference for both formulated diets.

### 4.4. Feeding of Zebrafish and Collection of Blood and Organs

**Figure 12:**
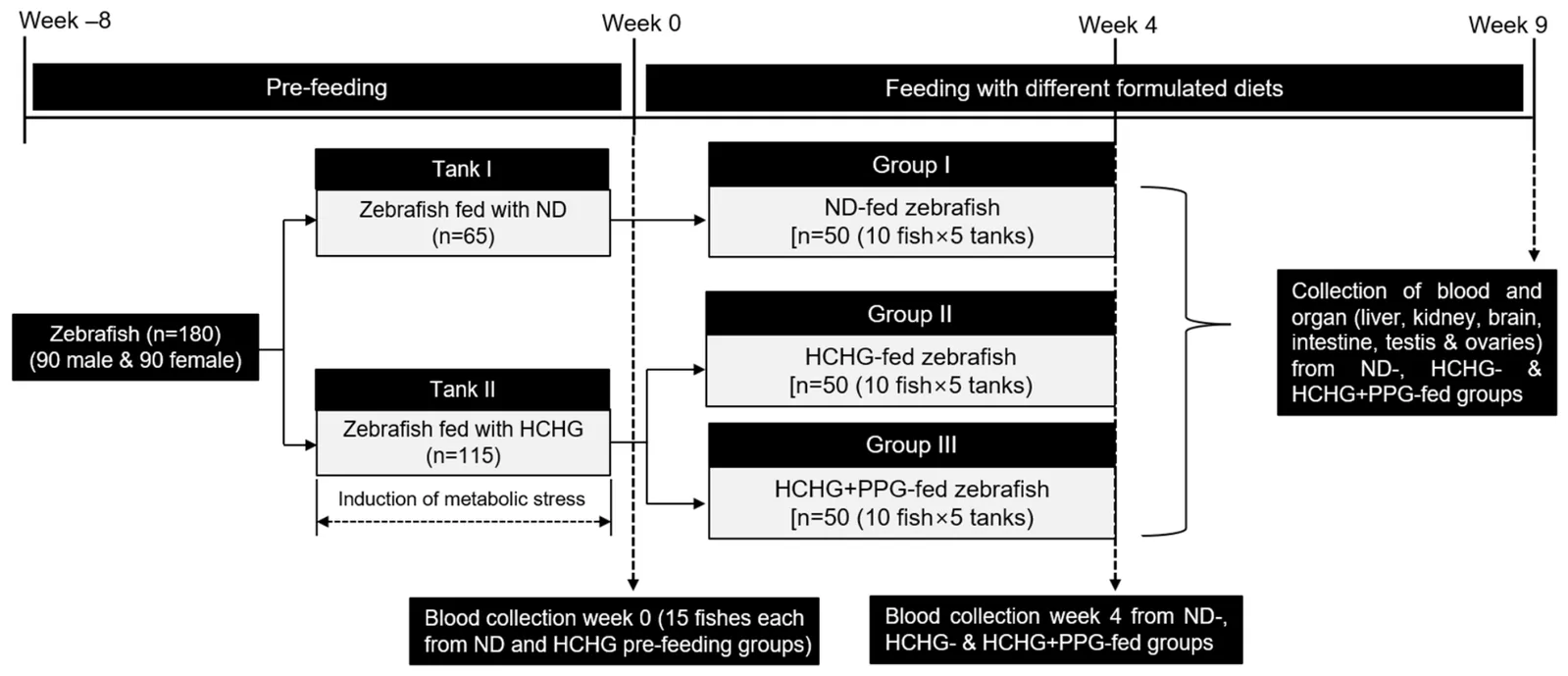
Layout of the experimental design. ND and HCHG, abbreviated for normal diet and high-cholesterol and high-galactose diet, respectively, whereas PPG represents a mixture of policosanol, phosphatidylserine, and *G. biloba* leaf extract.

At the beginning, 90 male and 90 female zebrafish (total 180) were maintained in the two different tanks. The zebrafish from both tanks were randomly transferred into tank I (*n* = 65, 33 females and 32 males), while the other zebrafish (*n* = 115, 57 females and 58 males) were transferred into tank II. The zebrafish in tank I were fed an ND diet for 8 weeks (pre-feeding), while zebrafish in tank II were fed an HCHG diet for 8 weeks (pre-feeding) to induce metabolic stress. After 8 weeks of pre-feeding, 15 zebrafish from tank I (8 females and 7 males) and tank II (7 females and 8 males) were removed and processed for body weight analysis and blood collection (week 0). The blood was processed for lipid quantification and biochemical analysis. After confirming the induction of metabolic stress (changes in body weight and blood lipid profiles and biochemical parameters) in the HCHG group compared to the ND group, the remaining fish from tanks I and II were further processed for the 9-week feeding, using the specialized diets. The remaining zebrafish (*n* = 50) from tank I continued to be fed an ND diet (Group I), whereas the remaining zebrafish (*n* = 100) from tank II were randomly assigned to two groups: HCHG (*n* = 50, 25 males and 25 females) (group II) and HCHG+PPG (*n* = 50, 25 males and 25 females) (group III). Zebrafish for each group (I to III) were randomly distributed into five independent tanks, i.e., each tank containing 10 fish, for a total of five replicates for each group (*n* = 10 fish × 5 tanks = 50) (Figure 12). Notably, each tank contains an equal sex distribution of 5 males and 5 females. Zebrafish in the respective groups were fed a specialized diet for 9 weeks, either ND, HCHG, or HCHG+PPG. A formulated diet of HCHG and HCHG+PPG (10 mg/zebrafish, containing ~6.3 mg of ND) was provided twice (9 am and 6 pm), equivalent to a cumulative diet of 200 mg/tank/day. Similarly, the ND diet (6.5 mg/zebrafish) was provided twice, equivalent to 130 mg/tank/day. Notably, all groups consumed nearly the same amount of the basal diet (i.e., ND).

The zebrafish (*n* = 50/group), housed in five tanks (10 fish/tank) as described earlier, were subsequently used for blood sample collection at week 4 and week 9. For blood collection at week 4, zebrafish (*n* = 25, five zebrafish from each tank) for the specialized group were sacrificed by hypothermic shock by immersing them in ice slush. In the euthanized zebrafish, an incision between the anal fin and caudal fin was made to obtain the blood [76], which was immediately collected by micropipette. Blood from the five zebrafish from a single tank was pooled in a single centrifuge tube and mixed with phosphate-buffered saline (PBS) with 1 mM EDTA in a 2:3 (*v*/*v*) ratio. The sample was processed for centrifugation (6000 rpm, for 10 min). The plasma was collected, diluted ~10 times (with PBS) to obtain a plasma protein concentration of 1 mg/mL, and preserved in a refrigerator for further analysis. Similarly, at week 9, all surviving zebrafish across the groups were euthanized and processed for blood collection using a similar methodology as described for the week 4 blood collection. Notably, zebrafish were fasted for 12 h before blood collection.

Different organs (liver, kidney, brain, intestine, testis and ovary) from the euthanized zebrafish were surgically removed under a stereo microscope (Motic SMZ 168; Hong Kong, China). The organs were preserved in 10% formalin. For the histological and blood biochemical analysis, researchers were blinded to the allocated groups. Samples were coded with a non-revealing alphabetical code prior to the analysis.

### 4.5. Biochemical Analysis of Plasma

The lipoprotein profile [total cholesterol (TC), triglycerides (TGs), high-density lipoprotein cholesterol (HDL-C)] and hepatic function biomarkers [aspartate aminotransferase (AST) and alanine aminotransferase (ALT)] in plasma were measured using commercial assay kits, following the manufacturers’ instructions. A detailed methodology has been provided in the Supplementary Method Section S1.

The plasma malondialdehyde (MDA), sulfhydryl content, paraoxonase (PON)-like activity and ferric ion reduction activity (FRA) were quantified following the earlier described method [49]. A detailed methodology is provided as Supplementary Method Section S2. The glucose level was measured with a digital blood glucose meter (Accu-Chek, Roche, Basel, Switzerland).

### 4.6. Microinjection of Plasma to Zebrafish Embryos

Zebrafish embryos were produced by using male and female zebrafish. Male and female zebrafish were separated from each other (~16 h) in the breeding tank using a physical divider. Afterwards, the divider was removed to allow the zebrafish to mate without any disturbance (~30 min) in the dark. Subsequently, the produced embryos were collected, rinsed with water, and maintained in a 0.01% (*w*/*v*) sea salt solution containing 1 μg/mL methylene blue. Zebrafish embryos were randomly segregated into four groups (*n* = 100/group). Embryos in group I were microinjected with 10 nL of PBS, while embryos in group II were microinjected with 10 nL of PBS containing 500 ng of CML. Embryos in groups III and IV were microinjected with 10 nL of plasma from HCHG and HCHG+PPG containing CML (500 ng), respectively. CML was used to induce oxidative stress, and the amount used was selected based on an earlier report, where 500 ng of CML showed substantial adverse effects on zebrafish embryos [45]. Embryos in different groups were examined during 72 h post-treatment for the detection of survivability; somite counts and teratogenic effects follow the OECD (test no. 236) 2025 guidelines [77]. Images for the developmental deformities were captured at 144 h post-treatment.

### 4.7. Dihydroethidium (DHE) and Acridine Orange (AO) Fluorescent Staining

Ten embryos (*n* = 10) from each group were placed in separate cells of a 24-well plate containing 0.2 mL solution of 30 μM DHE and 5 mg/mL of AO. After 30 min incubation, embryos were washed thoroughly three times with PBS. Finally, embryos were visualized under a fluorescent microscope (Nikon Eclipse TE2000, Tokyo, Japan) at the excitation wavelengths of 505 nm and 535 nm and the emission wavelengths of 585 and 615 nm for the detection of AO and DHE fluorescence, respectively.

### 4.8. Hematoxylin and Eosin (H&E), Oil Red O (ORO), and Cellular Senescence Staining

For the histological analysis, tissue from different organs (liver, kidney, brain, testis, and ovary) was individually submerged in frozen-section solution (Surgipath FSC22 solution, Leica, Nussloch, Germany). Immediately after embedding, the tissue blocks were stored in liquid nitrogen to solidify. The solidified tissue block was kept at −21 °C overnight and processed for tissue sectioning (7 μm thick) using a cryo-microtome (Leica, CM-1510S, Nussloch, Germany).

Histological changes in the tissue were assessed using H&E staining, following the previously described method [78]. For the ORO staining, the liver section (7 μm thick) was covered with ORO solution for 5 min at 60 °C. Subsequently, the section was rinsed with isopropanol and visualized under a microscope (Nikon, Tokyo, Japan).

Cellular senescence in tissue sections (7 μm thick) of liver, kidney, brain, testis and ovary was determined using senescence associated-β-galactosidase (SA-β-gal) staining [49]. Briefly, tissue section was covered with 0.1% of X-Gal (5-bromo)-4choloro-3-indolyl-β-galactopyranoside solution. After 15 h of incubation in a moist, cool atmosphere, the section was washed with water. After drying, the section was examined under a microscope to detect blue-stained senescent-positive cells.

### 4.9. Immunohistochemical (IHC) Analysis, Dihydroethidium (DHE) and Acridine Orange (AO) Fluorescent Staining

The interleukin (IL)-6 in the liver section was examined by IHC staining. A liver section (7 μm thick) was covered with 200× diluted IL-6-specific primary antibody (mouse IgG, ab9324, Abcam, Cambridge, UK). After 16 h of incubation in a cool, moist atmosphere, the section was washed twice with PBS. The section was treated with 1000×-diluted HRP-conjugated secondary antibodies as part of the EnVision HRP-polymer kit (K4001, Dako, Glostrup, Denmark). Finally, the section was developed using the chromogenic substrate provided with the kit (K4001, Dako, Glostrup, Denmark). The developed IHC section was visualized under the microscope.

The DHE and AO fluorescent staining of the tissue section (7 μm thick) was performed as described in Section 4.7.

### 4.10. Statistical Analysis

All experiments were performed at least in triplicate, and the results are presented as the mean ± standard error of the mean (SEM). The statistical comparisons between the groups were performed using one-way ANOVA, followed by Tukey’s post hoc analysis using the Statistical Package for the Social Sciences (SPSS, version 29, Chicago, IL, USA). Data normality was assessed prior to performing ANOVA.

## 5. Conclusions

The current study is one of the first reports documenting the beneficial effect of policosanol, phosphatidylserine and *G. biloba* leaf extract formulation (PPG) in preventing HCHG-induced metabolic stress and pathological changes in zebrafish. The PPG supplementation for 9 weeks mitigates HCHG-induced weight gain, hepatomegaly, and nephromegaly in zebrafish. Also, PPG supplementation restores the HCHG-disturbed plasma lipid profile and glucose levels and augments antioxidant status, thereby preventing fatty liver and damage to the kidney, ovary, testis, intestine and brain of zebrafish. The findings highlight the beneficial effect of PPG in mitigating HCHG-induced metabolic stress and multi-organ damage. Despite the promising outcomes in the zebrafish model, further validation in a mammalian model and clinical trials are required to assess the translational efficacy of PPG in preventing metabolic disorders in humans.

## Data Availability

The original contributions presented in this study are included in the article/Supplementary Materials. Further inquiries can be directed to the corresponding author.

## Supplementary Materials

The following supporting information can be downloaded at: https://www.mdpi.com/article/10.3390/ijms27177913/s1.
