# Supplementary material for: Nine-Week Co-Supplementation with Sugarcane Wax Alcohol (Policosanol), Phosphatidylserine, and Ginkgo biloba Leaf Extract Attenuates the Metabolic Dysfunction and Oxidative Stress in Blood and Vital Organs of Hyperlipidemic/Hyperglycemic Zebrafish
Source: Int J Mol Sci. 2026 Sep 4;27(17):7913. doi: 10.3390/ijms27177913 (PMC13566294; doi:10.3390/ijms27177913)
Supplement: Supplementary file 1 [file ijms-27-07913-s001.zip › ijms-4509802-supplementary.pdf]

Supplementary file

Supplementary Figure S1

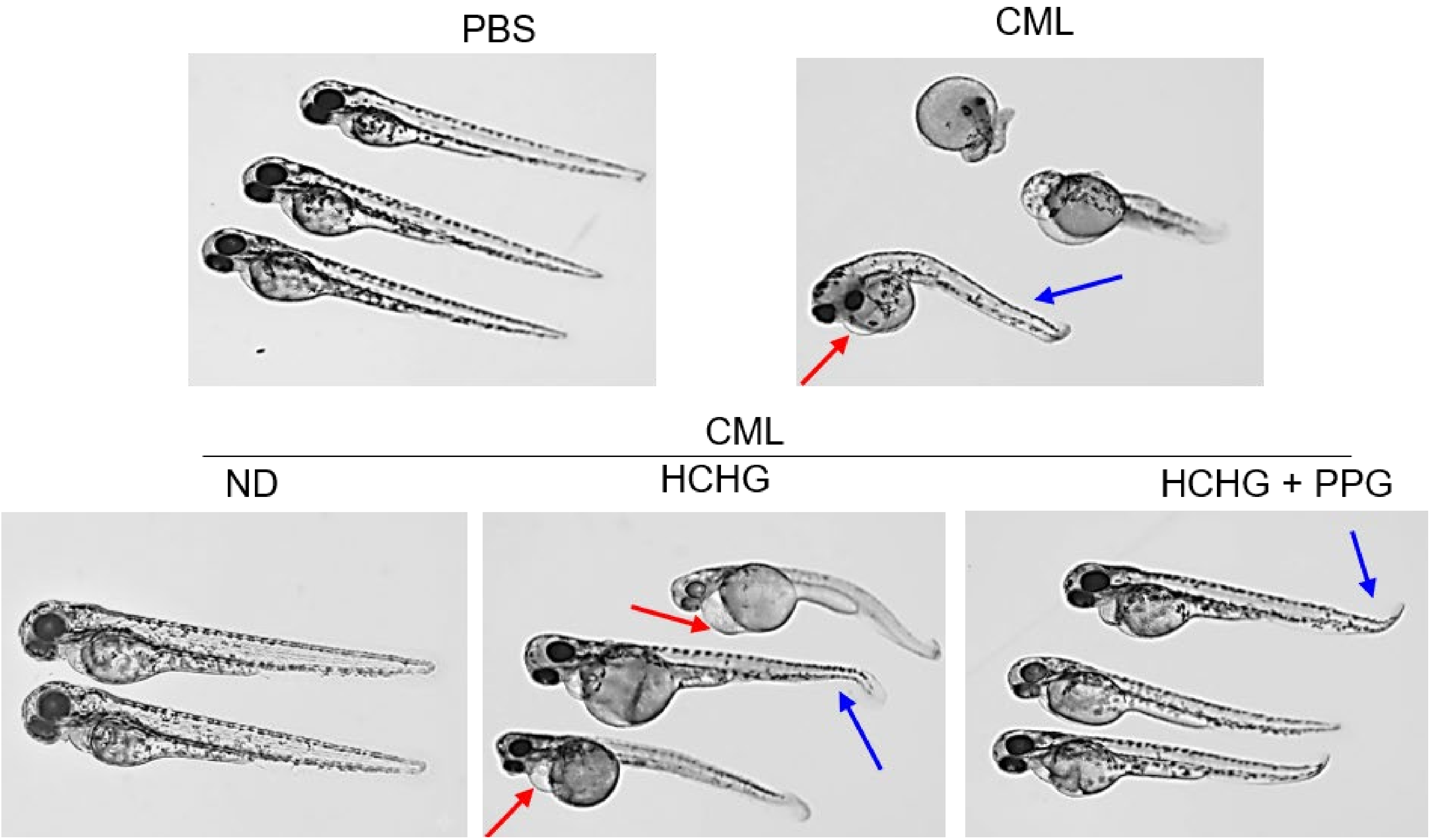

Supplementary Figure S1: Representative images of zebrafish embryos 72 h post treatment. Red and blue arrows highlight the pericardial edema and tail fin curvature, respectively. ND and HCHG, abbreviated for normal diet and high-cholesterol and high galactose diet, respectively, whereas PPG represents a mixture of policosanol, phosphatidylserine, and *Ginkgo biloba* leaf extract.

Supplementary Figure S2

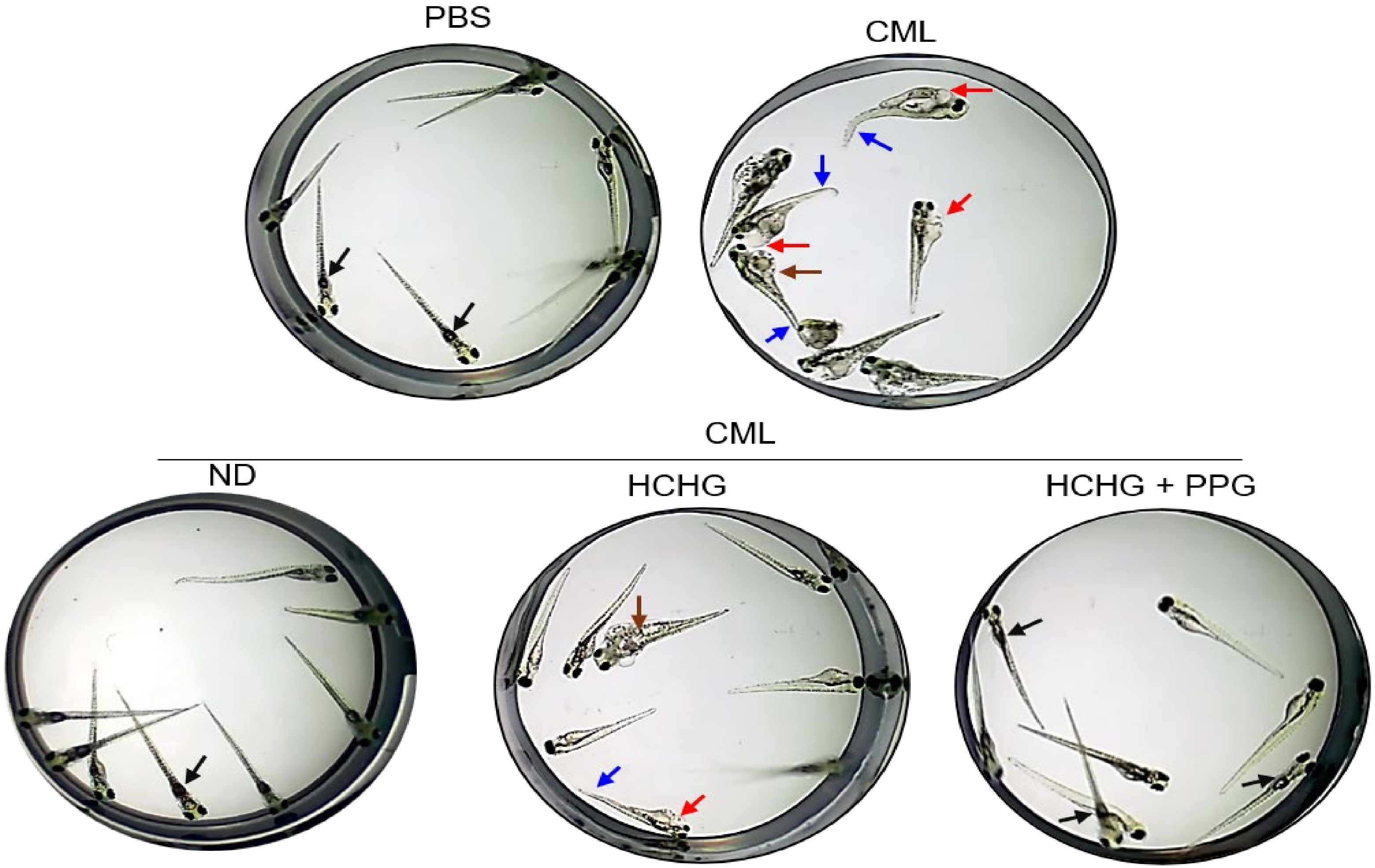

Supplementary Figure S2: Representative images of zebrafish embryos 144 h post treatment. Red arrows highlight the pericardial edema, blue arrows highlight tail fin curvature, brown arrows highlight yolk sac edema, black arrows depict the inflated swimming bladder. ND and HCHG, abbreviated for normal diet and high-cholesterol and high galactose diet, respectively, whereas PPG represents a mixture of policosanol, phosphatidylserine, and *Ginkgo biloba* leaf extract.

Supplementary Figure S3

RAYDEL
Health Confidence

Raydel Australia Pty Ltd
Level 1, building 1, 9-15 Chilvers Rd.
Thornleigh NSW 2120 Australia
Tel +61 2 9480 1300
Fax +61 2 9480 1399
A.B.N. 45 054 555 903
www.raydel.com.au
info@raydel.com.au

Product name: Policosanol

Batch #: 310030324

Date of Manufacture: 12/03/2024

Parameter	Results	Approved Limits
Color	Complies	Off white to cream
**Identity and Purity***		
1-tetracosanol (C_{24})	0.05 %	0.00 – 2.0 %
1-hexacosanol (C_{26})	3.34 %	3.0 –10.0 %
1-heptacosanol (C_{27})	0.82 %	0.1 - 3.0 %
1-octacosanol (C_{28})	60.02 %	60.0 –70.0 %
1-nonacosanol (C_{29})	0.55 %	0.1- 2.0 %
1-triacontanol (C_{30})	14.96 %	10.0 – 15.0%
1-dotriacontanol (C_{32})	8.24 %	5.0 - 10.0 %
1-tetratriacontanol (C_{34})	2.39 %	0.1 – 5.0 %
**Total (Purity*)**	**90.37 %**	**≥ 90 %**
**Other quality specifications**		
**Melting temperature**	81.3-83.0 °C	78.0 – 83.0 °C
**Loss on drying**	0.54 %	≤ 1.0 %
**Residue of ignition**	0.72 %	≤ 0.85 %
**Heavy metals (Pb, Cd, Hg)**	<0.000015 %	≤ 0.001 %
**Sodium content**	86.34 ppm	≤ 100 ppm
**Potassium content**	2905.41 ppm	≤ 4500 ppm
**Residual solvents**		
**Acetone**	≤ 0.03	≤ 0.03 g/kg
**Hexane**	≤ 0.005	≤ 0.005 g/kg
**Microbiological content**		
**Total Aerobic Microbial Count**	≤10	≤10^3 per g
**Yeast and mould**	≤10	≤10^2 per g
**Enterobacteria or Coliform count**	≤10	≤10^2 per g
***Staphylococcus aureus, Pseudomonas aeruginosa, Escherichia* coli, Candida albicans**	Absent	Absent in 1 g
Salmonella sp	Absent	Absent in 10 g
Observations:		
**References:**	* Manufacturer GC validated method, purity expressed as the total of high molecular weight alcohols	

Note about storage conditions: No special storage conditions are required. The substance has a shelf life of 5 years stored under ambient conditions of climatic Zones IV or II, as demonstrated in stability studies performed according to ICH guidelines.

Approved (_X_) Released (_X_) Rejected (___)

This COA is it reproduced from supplier's COA

Figure S3: Certificate of analysis and the composition of the policosanol used.

Supplementary Figure S4

VENBIOTECH (P) LTD

Office: Regd.off.10, Chokkanathar Street,
Karthikeyan Nagar, Maduravoyal,
Chennai – 600 095.

Factory: S.No:242/8a,b,c,Battai Road
Alathur –Sidco Industrial Area,
Thiruporur-Taluk,TamilNadu-603110.

CERTIFICATE OF ANALYSIS

Product Name : **PHOSPHATIDYLSERINE**

Batch Number : 20221204 Country of origin : India

Date of Manufacture : 04-December-2022 Date of Expiry : 03-December-2025

Source: Soybean Lecithin

Analysis	Specifications	Results
Description	Light yellow powder	Complies
Flavor/Taste	No peculiar smell	Complies
Impurity	No peculiar Impurity	Complies
Moisture	NMT 2.0 %	0.57%
Phosphatidylserine/(g/100g)	NLT70%	71.3%
Particle size	40-60mesh	Complies
Heavy Metals		Complies
- Arsenic	NMT 1.0 ppm	Complies
- Lead	NMT 1.0 ppm	Complies
- Cadmium	NMT 1.0 ppm	Complies
- Mercury	NMT 1.0 ppm	Complies
Hexane	NMT 5 ppm	Complies
Microbiological Analysis		
Total Aerobic Plate Count	NMT 10 cfu/g	Complies
Coliforms	Absent / 0.3 g	Absent
Escherichia coli	Absent / 1 g	Absent
Salmonella spp.	Absent /10 g	Absent
Staphylococcus aureus	Absent / 1 g	Absent

ANALYSED BY	QC CHEMIST	APPROVED BY	QC HEAD
NAME	V.RAJA	NAME	R.RAVI
DATE	December04, 2022	DATE	December04, 2022

Email Id: venu@venbiotech.in, accounting@venbiotech.in, venbiotec@gmail.com, venbiotech@yahoo.in,md1@venbiotech.in website url: www.venbiotech.in, Ph: +91 -44-23780113, Fax: +91-44-23780113, cell:+919940417693.

Figure S4: Certificate of analysis for phosphatidylserine.

Supplementary Figure S5

fine Pharma

Certificate of Analysis

**Product Name**	**Ginkgo Biloba Extract**		
**Botanical Source**	*Ginkgo biloba L.*	**Batch Number**	FN240619-02 ✓
**Plant Used**	Leaves	**Manufacturing date**	JUN 19,2024 ✓
**Solvents Used**	Water & Ethanol	**Expiration date**	JUN 18,2026 ✓
**Quantity**	2,000kg	**Country of Origin**	U.S.A

Item	Specification	Test Result	Method
**Physical & Chemical Data**			
Color	Brown Powder	Conforms	Visual
Odour	Characteristic	Conforms	Organoleptic
Appearance	Fine Powder	Conforms	Organoleptic
**Analytical Quality**			
Identification	Identical to R.S sample	Identical	HPTLC
Assay			
Total flavone Glycosides	≥24.0%	24.75%	HPLC
Kaempferol/Quercetin/	0.8-1.2	1.06%	HPLC
Ginkgolic Acid	≤5ppm	0.95ppm	HPLC
Sieve analysis	100% through 80mesh	Conforms	USP<786>
Loss on Drying	≤5.0%	3.30%	USP<731>
Total Ash	≤10.0%	1.60%	
**Contaminants**			
Lead(Pb)	≤1.0 mg/kg	Conforms	
Arsenic(As)	≤1.0 mg/kg	Conforms	
Cadmium(Cd)	≤1.0 mg/kg	Conforms	
Mercury(Hg)	≤0.1 mg/kg	Conforms	
Solvents Residue	≤5000 mg/kg	Conforms	
**Microbiological**			
Total Plate Count	≤10000cfu/g	120cfu/g	
Yeast & Mold	≤1000cfu/g	30cfu/g	
E.Coli	Negative	Conforms	
Coliforms	Negative	Conforms	
Salmonella	Negative	Conforms	

**Package**	25kg in Paper drums and two plastic-bags inside.
**Storage**	Store in normal temperature, keep away from heat and light.
**Conclusion**	Conforms to Standard

Analyzed by: Ailsa

Checked by: Yvonne

Fine Pharma INC @ Add: 1312 17th Street, Suite 692, Denver, CO 80202, United States
E-mail: sales@finepharma.com

Figure S5: Certificate of analysis for *Ginkgo Biloba* leaf extract.

Supplementary Figure S6

Original certificate of water quality

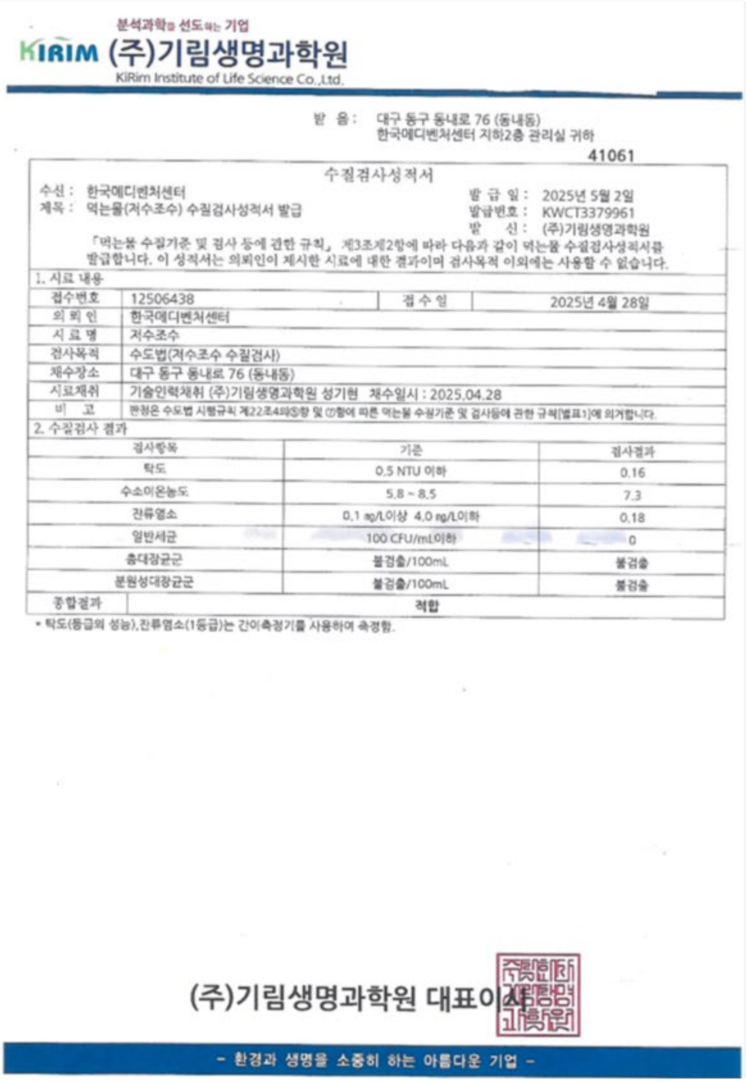
분석과학을 선도하는 기업

KIRIM (주)기림생명과학원

KiRim Institute of Life Science Co.,Ltd.

받 음: 대구 동구 동내로 76 (동내동)
한국메디벤처센터 지하2층 관리실 귀하

41061

수질검사성적서

수신 : 한국메디벤처센터

제목 : 먹는물(저수조수) 수질검사성적서 발급

발 급 일 : 2025년 5월 2일

발급번호 : KWCT3379961

발 신 : (주)기림생명과학원

「먹는물 수질기준 및 검사 등에 관한 규칙」 제3조제2항에 따라 다음과 같이 먹는물 수질검사성적서를 발급합니다. 이 성적서는 의뢰인이 제시한 시료에 대한 결과이며 검사목적 이외에는 사용할 수 없습니다.

1. 시료 내용

접수번호	12506438	접 수 일	2025년 4월 28일
의 뢰 인	한국메디벤처센터		
시 료 명	저수조수		
검사목적	수도법(저수조수 수질검사)		
채수장소	대구 동구 동내로 76 (동내동)		
시료채취	기술인력채취 (주)기림생명과학원 성기현 채수일시 : 2025.04.28		
비 고	판정은 수도법 시행규칙 제22조4의⑤항 및 ⑦항에 따른 먹는물 수질기준 및 검사등에 관한 규칙[별표1]에 의거합니다.		

2. 수질검사 결과

검사항목	기준	검사결과
탁도	0.5 NTU 이하	0.16
수소이온농도	5.8 ~ 8.5	7.3
잔류염소	0.1 ㎎/L이상 4.0 ㎎/L이하	0.18
일반세균	100 CFU/mL이하	0
총대장균군	불검출/100mL	불검출
분원성대장균군	불검출/100mL	불검출
종합결과	적합	

* 탁도(등급외 성능),잔류염소(1등급)는 간이측정기를 사용하여 측정함.

(주)기림생명과학원 대표이사

- 환경과 생명을 소중히 하는 아름다운 기업 -

Certificate of water quality (translated in English)

Drinking Water Quality Test Report
Recipient: Korea Mediventure Center
B2 Management Office
76 Dongnae-ro, Dong-gu, Daegu (Dongnae-dong), 41061
Issued by: Kirim Life Science Co., Ltd.
Issue Date: May 2, 2025
Report Number: KWCT3379961
Subject: Drinking Water (Reservoir Water) Quality Test Report

Basis of Issuance
This report is issued in accordance with Article 3, Paragraph 2 of the *Regulations on Drinking Water Quality Standards and Testing*.
It presents the results of the sample provided by the client. The report must not be used for purposes other than water quality testing.

1. Sample Information
- **Receipt Number:** 12506438
- **Receipt Date:** April 28, 2025
- **Client:** Korea Mediventure Center
- **Sample Name:** Reservoir Water
- **Purpose of Test:** Waterworks Act (Reservoir Water Quality Test)
- **Sampling Location:** 76 Dongnae-ro, Dong-gu, Daegu (Dongnae-dong)
- **Collected by:** Technical staff (Seong Ki-hyun, Kirim Life Science Co., Ltd.)
- **Sampling Date/Time:** April 28, 2025
- **Remarks:** Evaluation conducted in accordance with Article 22, Paragraph 4 of the Enforcement Rules of the Waterworks Act and Annex 1 of the *Regulations on Drinking Water Quality Standards and Testing.*

2. Test Results

Test Item	Standard	Result
Turbidity	≤ 5 NTU	0.16
pH (Hydrogen Ion)	5.8 – 8.5	7.3
Residual Chlorine	0.1 – 4.0 mg/L	0.18
General Bacteria	≤ 100 CFU/mL	0
Total Coliform Group	Not detected / 100 mL	Not detected
Fecal Coliform Group	Not detected / 100 mL	Not detected

Comprehensive Result: Suitable (Compliant)

Note: Turbidity (non-graded performance) and residual chlorine (Grade 1) were measured using a simplified measuring device.

Certification
Issued by: **Kirim Life Science Co., Ltd.** Representative Director
- A beautiful company that values environment and life -

Figure S6: Certificate of water quality analysis.

Supplementary Figure S7

Translated from Korean to English - www.onlinedoctranslator.com

A

Consumer Report on Functional Ingredients in Health Functional Foods

- Policosanol-Sugarcane Wax Alcohol (No. 2006-4), Raydel Korea Co., Ltd. -

Ministry of Food and Drug Safety

※ Data submitted by business operators, etc. among raw materials or ingredients not listed in the "Standards and Specifications for Health Functional Foods"

Please be advised that this information pertains to functional ingredients approved through review, has been prepared to aid consumer understanding, and cannot be used as a means of labeling, advertising, or promotion for the purpose of selling health functional foods.

Review Classification	Ingredients not listed in the "Standards and Specifications for Health Functional Foods"	O
	Addition of functional properties, intake amounts, and manufacturing standards for ingredients notified in the "Standards and Specifications for Health Functional Foods"	-

Raw Material Overview

Functional ingredient name (Recognition Number)	· Policosanol-Sugarcane Wax Alcohol (No. 2006-4)
Functional content	· May help improve blood cholesterol levels<br>· May help regulate blood pressure
Daily intake	· Blood cholesterol: 5–20 mg as policosanol-sugarcane wax alcohol<br>· Blood pressure regulation: 20 mg as Policosanol-Sugarcane Wax Alcohol
When consuming caution	· Pregnant women, breastfeeding mothers, adolescents, and children should avoid consumption.<br>· Individuals taking cardiovascular medication, those with blood clotting disorders, or those scheduled for surgery should consult a specialist.<br>· If adverse effects occur, discontinue use and consult a healthcare professional.

Reference content

* Relevant Regulation: Article 15 of the "Regulations on the Recognition of Functional Ingredients and Standards and Specifications for Health Functional Foods"

1. Standards and Specifications

○ Policosanol-Sugarcane Wax Alcohol (No. 2006-4) is sugarcane (scientific name:*Saccharum officinarum*L., parts used: leaves, stems) as a raw material obtained by crushing, pressing, extracting, heating, saponifying, purifying, heating, purifying, and drying,

- Characteristics, Function (Indicator) Components·Standards for hazardous substances (heavy metals, microorganisms, residual solvents) have been established.

* 1-tetracosanol: 0.1-20.0 mg/g, 1-hexacosanol: 30.0-100.0 mg/g, 1-heptacosanol: 1.0-30.0 mg/g 1-octacosanol: 600.0-700.0 mg/g, 1-nonacosanol: 1.0-20.0 mg/g, 1-triacontanol: 100.0-150.0 mg/g 1-dotriacontanol: 50.0-100.0 mg/g, 1-tetratriacontanol: 1.0-50.0 mg/g

B

2. Safety

○ Basis for intake (domestic/international recognition/usage status, etc.), safety information data, intake assessment data (with domestic/international distributed products)

As a result of a comprehensive evaluation of the average intake comparison), toxicity test data, safety data from human clinical trials, etc.,

- of policosanol-sugar cane wax alcohol (No. 2006-4)The daily intake (5–20 mg/day) is at a safe level.was

3. Functionality

1) May help improve blood cholesterol levels

※ Approved in March '06

○ Basic research such as human clinical trials and animal trialsOverall evaluationAs a result, policosanol-sugar cane wax alcoholDaily intake (5~20 mg/day)atEvidence of functionalityThere was

- (Human clinical trials)Policosanol-Sugarcane Wax alcohol(5~20 mg/day)As a result of consuming for 8 weeks, total cholesterol(Total cholesterol), LDL-cholesterol(LDL-cholesterol)This significantly decreases, and HDL-cholesterol (HDL-cholesterol)this It increased significantly

- (Basic Research)animal testing(white rabbit, monkey, wistar rat)Policosanol-Sugarcane Wax As a result of oral administration of alcohol for 4 and 30 days, total cholesterol(Total cholesterol), LDL-cholesterol, indicators related to peroxide formation(MDA)Since it was significantly reduced, the basic research explained the mechanism of action. Scientifically supported by the results of human clinical trials

2) May help control blood pressure

※ Approved in July '19

○ Basic research such as human clinical trials and animal trialsOverall evaluationAs a result, policosanol-sugar cane wax alcoholDaily intake (20 mg/day)atEvidence of functionalityThere was

- (Human clinical trials)Policosanol-Sugarcane Wax Alcohol in 76 adult men and women aged 18 to 65 with prehypertension(20 mg/day)As a result of consuming for 12 weeks, systolic and diastolic blood pressure, average Arterial pressure, etc. decreased significantly

- (Basic Research)animal testing(Hypertension-induced animal model)Policosanol-sugar cane wax alcohol from As a result of 8 weeks of oral administration, systolic and diastolic blood pressure, blood flow velocity, blood flow volume, and aldosterone(Aldosterone), Lipoprotein composition(HDL-cholesterol, LDL-cholesterol, HDL-C/TC ratio)Significant differences appearing in the back Basic research explains the mechanism of action and scientifically supports the results of human clinical trials.

Figure S7 (A&B): A recommended dose of policosanol suggested by Ministry of Food and Drug Safety (MFDS).

Supplementary Figure S8

A

Translated from Korean to English - www.onlinedoctranslator.com

2-29 Phosphatidylserine

1) Manufacturing Standards

(1) Raw material: Soybean lecithin

(2) Manufacturing method

: The raw materials of (1) above are L-serine and enzyme (phospholiphase) It must be manufactured by reacting, extracting with water, ethanol (including water-ethanol mixtures), acetone, or hexane, and purifying.

(3) Content of functional ingredient (or indicator ingredient): Must contain 380 mg/g or more of phosphatidylserine.

2) Specifications

(1) Appearance: It must have its own color and flavor and be free from off-flavors and odors.

(2) Phosphatidylserine

(a) Raw material products: Indicated amount or more (b) Final products: 80 ~ 120% of the indicated amount

(3) Residual solvent (only if the relevant solvent was used) (a) Hexane (mg/kg): 5.0 or less

(b) Acetone (mg/kg): 10.0 or less

(4) heavy metals

(a) Lead (mg/kg): 1.0 or less (b) Cadmium (mg/kg): 1.0 or less (c) Mercury (mg/kg): 1.0 or less (d) Arsenic (mg/kg): 1.0 or less

(5) Coliform bacteria: negative

3) Requirements for the final product

(1) Functional content

: Improvement of cognitive function declined due to aging · Protection from skin damage caused by UV rays May help maintain skin health and moisturize the skin

(2) Daily intake: 300 mg as phosphatidylserine

B

(3) Precautions when consuming

Pregnant and nursing women should exercise caution when consuming.

Excessive consumption may cause gastrointestinal disorders or insomnia.

**Notice No. 2025-11 (March 5, 2025), Effective Date (January 1, 2026)**
(3) Precautions when consuming<br>(a) Pregnant and nursing women should avoid consumption.<br>(b) If adverse events occur, discontinue use and consult a healthcare professional.

4) Test method

(1) Appearance: Section 4.2-7 Appearance Test Method

(2) Phosphatidylserine: 4.3-70 Phosphatidylserine

(3) Residual solvent: Refer to [Appendix 4]

(4) Lead, cadmium, mercury, arsenic: See [Appendix 4]

(5) Coliforms: Refer to [Appendix 4]

Figure S8 (A&B): A recommended dose of phosphatidylserine suggested by Ministry of Food and Drug Safety (MFDS).

Supplementary Figure S9

Translated from Korean to English - www.onlinedoctranslator.com

A

2-13 Ginkgo leaf extract

1) Manufacturing Standards

(1) Raw material: Ginkgo tree (*Ginkgo biloba*) leaves

(2) Manufacturing method

: After crushing the raw material of (1) above, use it with alcohol (including a mixture of water and alcohol).

It must be manufactured by extraction, purification, concentration, and filtration.

(3) Content of functional components (or indicator components)

: Contains 240 mg/g or more of flavonol glycosides

It must be present, and the ratio of quercetin to kaempferol must be 0.8 to 1.2

(quercetin : kaempferol = 1 : 0.8 to 1.2).

2) Specifications

(1) Appearance: It must have its own color and flavor and be free from off-flavors and odors.

(2) Flavonol glycosides

(a) Raw material products: Indicated amount or more (b)

Final products: 80 ~ 120% of the indicated amount

(3) Ginkgolic acid (mg/kg): 5.0 or less

(4) heavy metals

(a) Lead (mg/kg): 1.0 or less (b)

Cadmium (mg/kg): 1.0 or less (c)

Mercury (mg/kg): 1.0 or less (d)

Arsenic (mg/kg): 1.0 or less

(5) Coliform bacteria: negative

3) Requirements for the final product

(1) Functional content: May help improve memory and blood circulation

(2) Daily intake: 28 ~ 36 mg as flavonol glycosides

(3) Precautions when consuming

Pregnant women, nursing mothers, children, and patients before and after surgery should exercise caution when consuming.

Exercise caution when consuming if taking medication (anticoagulants).

B

Notice No. 2025-11 (March 5, 2025), Effective Date (January 1, 2026)

(3) Precautions when consuming

(a) Children, pregnant women, and breastfeeding mothers should avoid consumption.

(b) Consult a specialist before and after surgery, or when taking medication (anticoagulants). (c) If adverse events occur, discontinue use and consult a specialist.

4) Test method

(1) Appearance: Section 4.2-7 Appearance Test Method

(2) Flavonol glycosides: 4.3-61 Flavonol glycosides

(3) Ginkgolic acid: 4.3-62 Ginkgolic acid

(4) Lead, cadmium, mercury, arsenic: See [Appendix 4]

(5) Coliforms: Refer to [Appendix 4]

Figure S9 (A&B): A recommended dose of *Ginkgo biloba* leaf suggested by Ministry of Food and Drug Safety (MFDS).

Section S1.

1. Method to quantify plasma lipoproteins and hepatic function biomarkers

The plasma total cholesterol (TC) and triglycerides (TGs) were determined using commercial assay kits (cholesterol, AM 202-K, and TGs, AM 157-K, Asan Pharmaceutical, Hwasung, Republic of Korea) as per the method suggested by the suppliers. In brief, 5 μL serum was mixed with 200 μL reaction mixture (supplied with a commercial assay kit) for the TC analysis. The content was incubated at 37°C for 10 min, resulting in a red-colored product quantified by adsorption at 490 nm (Microplate reader, iMark™ Bio-Rad, Hercules, CA, USA).

Similarly, 5 μL serum was mixed with a 200 μL of TGs-specific reaction mixture (supplied with a commercial assay kit) for TGs analysis. The content was incubated for 10 min at 37°C, and the formed colored product was quantified by taking adsorption at 490 nm.

For HDL-C analysis, serum was mixed in an equal ratio with the separation solution (supplied with a commercial assay kit), followed by centrifugation at 3,000 rpm for 10 min at 25°C. The supernatant (20 μL) was collected and blended with a 200 μL reaction mixture (supplied with a commercial assay kit). After 10 min incubation at 37°C, red color intensity corresponding to HDL-C was quantified by taking absorption at 490 nm (Microplate reader, iMark™ Bio-Rad, Hercules, CA, USA). The LDL-C level was quantified using the Friedewald equation:

LDL-C=TC−HDL-C−(TG/5)

The commercial diagnostic kit (AM102K and AM103-K, Asan Pharmaceutical, Hwasung, Republic of Korea) was used to quantify aspartate transaminase (AST) and alanine transaminase (ALT) levels in the plasma, following the instructions suggested by the manufacturers. Briefly, 5 μL of plasma was combined with 250 μL of either AST or ALT-specific solution, as supplied in the diagnostic kit. Following a 30 min incubation for AST or 60 min incubation of ALT at 37°C, the mixture was then blended with 250 μL of the respective coloring reagent (AST or ATL-specific, provided in the diagnostic kit). After a subsequent 20 min incubation at room temperature, 250 μL of 0.4 N NaOH was introduced to halt the reaction. Finally, the AST and ATL were quantified by measuring absorbance at 490 nm (Microplate reader, iMark™, Bio-Rad, Hercules, CA, USA).

Section S2.

2. Malondialdehyde (MDA), sulfhydryl group, ferric ion reduction (FRA) activity and paraoxonase (PON) activity

The blood malondialdehyde (MDA) level was quantified by mixing plasma sample (20 μL, equivalent to 1 mg/mL protein) with trichloroacetic acid (50 μL, 0.2 mg/μL, pH 1.4) and thiobarbituric acid (100 μL, 6.7 μg/μL, pH 2.3). Following a 10-min incubation at 95 °C, the absorbance at 560 nm was recorded.

The sulfhydryl group was quantified by mixing 60 μL of plasma (1 mg/mL protein) with 60 μL of 5,5′-dithio-bis-(2-nitrobenzoic acid) (DTNB) (4 mg/mL). After 12 hr incubation at room temperature, absorbance 412 nm was determined, and sulfhydryl groups were quantified utilizing 13,600 M^{-1} cm^{-1} extinction coefficient (e) of DTNB.

To assess ferric ion reduction (FRA) capacity, 20 μL of the plasma (1 mg/mL equivalent protein) was mixed with 180 μL of FRA reagent (prepared by blending 10 mL of

acetate buffer (0.2M, pH 3.6) with 1.25 mL each of 2,4,6-tripridyl-S triazin (10 mM, pH 1.9) and $FeCl_3$ (20 mM, pH 2.1). After incubating the mixture at RT for 60 min, absorbance was measured at 593 nm. The results were quantified in μM ferric equivalents based on a ferrous sulfate standard curve.

For paraoxonase activity 20 μL of plasma (1 mg/mL equivalent protein) was mixed with 180 μL of buffer (pH 8.3) [Tris-HCl (90 mM), NaCl (3.6 mM), $CaCl_2$ (90 mM)] containing the paraoxon-ethyl substrate (0.55 M). After 60 min incubation at 25°C, an absorbance (415 nm) was recorded using Microplate reader (Model, iMark™ S.N 21275, Bio-Rad, Hercules, CA, USA) to quantify the production of *p*-nitrophenol, a hydrolysis product of paraoxon-ethyl. Results are expressed as μU/L/min employing the extinction coefficient 17,000 $M^{-1}cm^{-1}$ for *p*-nitrophenol.
